# Unique Members of the Adipokinetic Hormone Family in Butterflies and Moths (Insecta, Lepidoptera)

**DOI:** 10.3389/fphys.2020.614552

**Published:** 2020-12-17

**Authors:** Heather G. Marco, Petr Šimek, Gerd Gäde

**Affiliations:** ^1^Department of Biological Sciences, University of Cape Town, Rondebosch, South Africa; ^2^Biology Centre, Czech Academy of Sciences, České Budějovice, Czechia

**Keywords:** butterflies and moths, Lepidoptera, adipokinetic hormone, mass spectrometry, primary structure, neuropeptides, biological assays

## Abstract

Lepidoptera is amongst one of the four most speciose insect orders and ecologically very successful because of their ability to fly. Insect flight is always aerobic and exacts a high metabolic demand on the animal. A family of structurally related neuropeptides, generically referred to as adipokinetic hormones (AKHs), play a key role in triggering the release of readily utilizable fuel metabolites into the hemolymph from the storage forms in the fat body. We used mass spectrometry to elucidate AKH sequences from 34 species of Lepidoptera and searched the literature and publicly available databases to compile (in a phylogenetic context) a comprehensive list of all Lepidoptera sequences published/predicted from a total of 76 species. We then used the resulting set of 15 biochemically characterized AKHs in a physiological assay that measures lipid or carbohydrate mobilization in three different lepidopteran species to learn about the functional cross-activity (receptor-ligand interactions) amongst the different butterfly/moth families. Our results include novel peptide structures, demonstrate structural diversity, phylogenetic trends in peptide distribution and order-specificity of Lepidoptera AKHs. There is almost an equal occurrence of octa-, nona-, and decapeptides, with an unparalleled emphasis on nonapeptides than in any insect order. Primitive species make Peram-CAH-II, an octapeptide found also in other orders; the lepidopteran signature peptide is Manse-AKH. Not all of the 15 tested AKHs are active in *Pieris brassicae*; this provides insight into structure-activity specificity and could be useful for further investigations into possible biorational insecticide development.

## Introduction

Butterflies are esthetically beautiful and, hence, not only collected by many laymen enthusiasts but are also displayed for the general public in “butterfly houses.” The negative impact of certain Lepidoptera are also known, e.g., the larvae of the fall and African armyworms (*Spodoptera frugiperda* and *Spodoptera exempta*; superfamily: Noctuoidea) damage crops that are the main staple food sources of people (Baudron et al., 2019); the Indian meal moth larvae (*Plodia interpunctella*; superfamily: Pyraloidea) devour stored food products, and species of the genera *Tinea* and *Tineola* (superfamily: Tineoidea) feed on woolen textiles (Basuk and Behera, 2018). In addition, toxins and/or the hairs/bristles of larvae from certain lepidopteran species cause severe medical problems such as urticarial dermatitis, allergies, and asthma (Donato et al., 1998; Marzano et al., 2020).

There are, however, several other traits that are known of Lepidoptera to classify them as “beneficial” for humankind. Many species are known pollinators (even specialist pollinators) (Veerman and Van Zon, 1965; Aker and Udovic, 1981; Rader et al., 2016). Further beneficial traits of Lepidoptera are the following, to name a few that are relevant to South Africa: (i) economically important sericulture not only with the domesticated *Bombyx mori* (superfamily: Bombycoidea) but also wild silk production using the African wild silk moth *Gonometa postica* (superfamily: Lasiocampoidea) (Teshome et al., 2014). (ii) biological weed control, e.g., larvae of the cactus moth, *Cactoblastis cactorum* (superfamily: Pyraloidea), control invasive *Opuntia* species introduced to Australia and South Africa (Anneke and Moran, 1978). (iii) Larvae of various species are sought-after delicacies for human populations in several countries worldwide: the high demand for Mopane worm in southern Africa (Emperor moth *Gonimbrasia belina*; superfamily: Bombycoidea) drives a lucrative market for women who collect the larvae in a sustainable manner (Sekonya et al., 2020).

**FIGURE 1:**
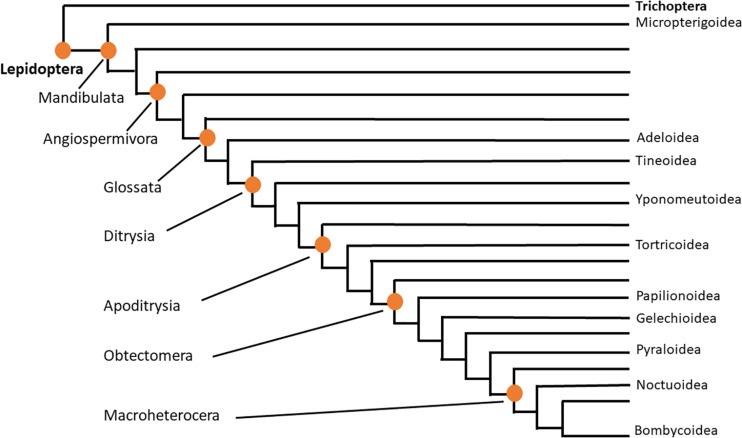
Evolutionary tree of Lepidoptera. Adapted from Mitter et al. (2017) and Kawahara et al. (2019), this condensed tree reflects only the superfamilies that are dealt with in the current study. The two orders are indicated in bold letters; clades within the order Lepidoptera are indicated on the left; lepidopteran superfamilies covered in the current study are shown on the right.

Lepidoptera is, thus, clearly a diverse order with “beneficial” and “pest” insect status. Linked to this is the high number of extant butterfly/moth species (almost 160,000 described species) that puts Lepidoptera amongst one of the four most speciose insect orders besides Coleoptera, Hymenoptera, and Diptera (Grimaldi and Engel, 2005; van Nieukerken et al., 2011). The importance of a robust phylogenetic treatment of Lepidoptera to provide a framework for interpreting and understanding behavioral, metabolic and environmental processes, for example, in an evolutionary context, has interested scientists for a long time (a few recent examples are: Kristensen et al., 2007; Regier et al., 2013; Mitter et al., 2017). The latest and most comprehensive phylogenomic study takes the transcriptome and genome of 186 species of 34 of the 43 superfamilies into account and interprets 2098 protein-coding genes (Kawahara et al., 2019). Figure 1 is a simplified, condensed version of the phylogenetic tree of Lepidoptera as proposed by Mitter et al. (2017) and Kawahara et al. (2019) and is based on the superfamilies investigated in the current study, thus it serves as orientation. The afore-mentioned studies all find monophyly of Lepidoptera, and the order Trichoptera (caddisflies) is a sister group, thus the closest relative. The largest clade of the Lepidoptera is the Ditrysia comprising 98% of the order’s species. The “non-ditrysian” Lepidoptera are identified as “primitive moths,” whose adults have mandibulate mouthparts in place of a proboscis, and consist of 14 superfamilies with the Micropterigoidea, that feed on detritus and bryophytes, as sister group to the remaining Lepidoptera (Figure 1). Tineoidea (bagworm moth and clothes moths) and the sister group Yponomeutoidea (best known example is the pest diamond back moth *Plutella xylostella* from the family Plutellidae) are at the base of the Dytrisia clade within which the group Apodytrisia resides which consists, *inter alia*, of the species-rich superfamily Tortricoidea (leaf roller moths, such as the pest species codling moth *Cydia pomonella*). Within Apodytrisia the next large clade is the Obtectomera (Figure 1), harboring all butterflies (Papilionoidea) and also the Pyraloidea (snout moths such as the meal moth genus *Plodia* or the cactus moth *C. cactorum*). Within the Obtectomera the clade Macroheterocera has been coined, consisting of the well-known and species-rich superfamilies of Noctuoidea (the owlet moths such as the fall army worm *S. frugiperda* or the corn earworm moth *Helicoverpa zea*) at the base, Geometroidea and as most advanced superfamily, the Bombycoidea with hawk moth, emperor moth and silk moth (Mitter et al., 2017; Kawahara et al., 2019; see Figure 1).

Insects were the first to have evolved sustained powered flight; fast fliers and long distance fliers are found amongst lepidopteran species with skippers (superfamily: Hesperioidea) clocking speeds of about 60 km h^–1^ through the air^1^ and migratory flights – an important strategy for survival and reproduction – observed and studied in tortricid and noctuid pest species (Schumacher et al., 1997; Jiang et al., 2011; Zheng et al., 2011; Stokstad, 2017; Guo et al., 2020), in nymphalids (superfamily: Papilionoidea) (Brattström et al., 2010; Stefanescu et al., 2013; Chapman et al., 2015; Agrawal, 2017), as well as in a number of sphinx moths (superfamily: Bombycoidea; Reinhardt and Harz, 1989). From an energetic point of view, flight is the most demanding activity of insects, always based on aerobic metabolism, and oxygen consumption can be elevated above resting values by more than 100-fold (Kammer and Heinrich, 1978; Gäde, 1992). The major storage site for metabolic fuel is the fat body – triacylglycerides and/or glycogen stores are mobilized by peptide neurohormones of the so-called adipokinetic hormone (AKH) family to replenish the fast diminishing fuels in the contracting flight muscles during wing beating (Gäde, 1992). Zebe (1954) measured a respiratory quotient (RQ) of 0.7–0.8 in most studied butterfly and moth species and deduced the usage of fat as flight substrate. This was corroborated by measuring maximal activities of specific enzymes of major energetic pathways (Beenakkers, 1969; Crabtree and Newsholme, 1972) or measuring the metabolic rate plus quantifying the metabolites (lipids, glycogen, and trehalose) in the fall army worm moth *S. frugiperda* (Nayar and van Handel, 1971; van Handel and Nayar, 1972). On the other hand, there are also reports on the participation of carbohydrates in flight metabolism of Lepidoptera. Whereas unfed adults of the tobacco hornworm moth *Manduca sexta* used almost exclusively fat for flights (Ziegler and Schulz, 1986b), carbohydrates play a role in the same species during the initial phase of pre-flight warm-up (Joos, 1987). In a diurnal nectar-feeding hawkmoth (*Amphion floridensis*) RQ measurements established that fed moths primarily oxidized carbohydrates, whereas starved moths burnt almost only fats (O’Brien, 1999). In a short-distance flyer like the nymphalid Glanville fritillary butterfly (*Melitaea cinxia*) that flies about 500 m in 2 h as median maximal flight distance (Ovaskainen et al., 2008), an RQ of about 1.0 has been measured for flights of 10 min, thus indicating that carbohydrates are exclusively used (Haag et al., 2005).

Peptides of the AKH family are responsible for regulating mobilization of stored metabolites especially during intense locomotory activity such as flight, swimming, or running in insects (Gäde, 1997; Marco and Gäde, 2020). These peptides are functionally comparable to vertebrate glucagon; structurally, however, AKH peptides and their cognate G-protein coupled receptors are related to the vertebrate gonadotropin releasing hormone (GnRH) system and together with two other insect neuropeptide systems, viz. corazonin (Cor) and its receptor, as well as AKH/corazonin-related peptide (ACP) and its receptor, comprise a large peptide superfamily (Hansen et al., 2010; Gäde et al., 2011; Roch et al., 2011). AKHs are synthesized and released from the neurohemal corpus cardiacum (CC). By primary structure the peptides are characterized by a chain length of 8–10 amino acid residues with post-translationally modified termini: a pyroglutamate residue at the N-terminus and a carboxy-amidation at the C-terminus, thus making the peptide insusceptible to digestion by exopeptidases. Other positions in the molecule show little variation when comparing multiple sequences: residue 2 from the N-terminus is either an aliphatic leucine, isoleucine, valine, or an aromatic phenylalanine or tyrosine; residue 3 is either threonine or asparagine, while residue 4 may be one of the two aromatic amino acids phenylalanine or tyrosine, and residue 5 may be either threonine or serine; a fair variety of amino acids can take up residues 6, 7, and 10, while residues 8 and 9 are always tryptophan and glycine, respectively (Gäde, 2009). More than 80 members of this peptide family are now known by sequence (Gäde, 2009; Marco and Gäde, 2020).

In Lepidoptera the first AKH that was completely structurally elucidated came from the CC of the tobacco hornworm moth, *M. sexta* (Family: Sphingidae) – the nonapeptide is called Manse-AKH (pELTFTSSWG amide; Ziegler et al., 1985), and it functions as a true adipokinetic, thus lipid-mobilizing, hormone in the adult moth and regulates carbohydrate metabolism in the larval stage (Ziegler et al., 1990). Since then, Manse-AKH was shown to be synthesized in the CC of a number of other butterflies and moths (for family and species details, see Table 1). The gene for Manse-AKH was also the first AKH gene that was cloned (Bradfield and Keeley, 1989). During the first separation attempts of biologically active AKH hormones in *M. sexta*, it became clear that activity was separated into two peaks (Ziegler and Gäde, 1984), one of which was the later identified Manse-AKH. It took, however, 28 years for the second *M. sexta* AKH peak to be sequenced by mass spectrometric methods as a decapeptide that was then found present in other sphingid moths as well (Manse-AKH-II, pELTFSSGWGQ amide; Weaver et al., 2012).

**TABLE 1:** The distribution of AKH peptides in the Lepidoptera, to date: primary sequence and calculated protonated mass.

**Higher taxonomy**	**Family**	**Species**	**AKH name**	AKH sequence*	**(M + H)^+^**	References**
Glossata, Adeloidea	Adelidae	*Nemophora (=Nematopogon) pilella*	Peram-CAH-II	pELTFTPNWa	988.4887	T011091201
Ditrysia, Tineoidea	Psychidae	*Eumeta japonica*	NOVEL 1	pELTFTSNWGSa	1122.5214	GBP11607.1; Kono et al., 2019
Ditrysia, Yponomeutoidea	Pluttellidae	*Plutella xylostella*	Lacol-AKH Peram-CAH-II	pELTFTSSWGGa pELTFTPNWa	1065.5000 988.4887	XP_011565387.1 XP_011555237.1
Apoditrysia, Tortricoidea	Tortricidae	*Cryptophlebia peltastica*	Lacol-AKH Peram-CAH-II	pELTFTSSWGGa pELTFTPNWa	1065.5000 988.4887	This study
		*Thaumatotibia leucotreta*	Lacol-AKH	pELTFTSSWGGa	1065.5000	This study
		*Cydia pomonella*	Lacol-AKH Peram-CAH-II	pELTFTSSWGGa pELTFTPNWa	1065.5000 988.4887	This study; Garczynski et al., 2019
Obtectomera, Papilionoidea	Papilionidae	*Papilio machaon*	Manse-AKH	pELTFTSSWGa	1008.4785	KPJ15512.1
		*Papilio xuthus*	Manse-AKH	pELTFTSSWGa	1008.4785	KPI97662.1
		*P. demodocus*	Manse-AKH ^#^(Vanca-AKH)	pELTFTSSWGa pELTFTSSWGGK-OH	1008.4785 1194.5790	This study
		*Papilio polytes*	Manse-AKH	pELTFTSSWGa	1008.4785	PpolytesGene0002562.mrna1
		*Papilio glaucus*	Manse-AKH	pELTFTSSWGa	1008.4785	Pgl722.20mrna
		*P. memnon*	Manse-AKH	pELTFTSSWGa	1008.4785	This study
		*Parides arcas*	Manse-AKH	pELTFTSSWGa	1008.4785	TO111202337
	Hesperiidae	*Lerema accius*	Manse-AKH	pELTFTSSWGa	1008.4785	Lac1208.8mRNA
	Pieridae	*Gonepteryx rhamni*	Manse-AKH	pELTFTSSWGa	1008.4785	This study
		*Pieris napi*	Manse-AKH	pELTFTSSWGa	1008.4785	PIENAPT00000010987
		*Pieris rapae*	Manse-AKH	pELTFTSSWGa	1008.4785	PIERAPT00000002073
		*P. brassicae*	Manse-AKH Piebr-AKH	pELTFTSSGWa pELTFSSGWa	1008.4785 907.4308	Marco and Gäde, 2017
		*Leptidea sinapis*	Manse-AKH	pELTFTSSWGa	1008.4785	VVC88225.1
		*Phoebis sennae*	Manse-AKH	pELTFTSSWGa	1008.4785	Pse4122.11
		*Catopsilia florella*	Manse-AKH	pELTFTSSWGa	1008.4785	This study
	Lycaenidae	*Polyommatus icarus*	Peram-CAH-II	pELTFTPNWa	988.4887	T011091239
		*Calycopis cecrops*	Manse-AKH	pELTFTSSWGa	1008.4785	Cce2042.16mRNA
	Nymphalidae	*Cethosia biblis*	Manse-AKH Piebr-AKH	pELTFTSSWGa pELTFSSGWa	1008.4785 907.4308	This study
		*Heliconius melpomene*	Manse-AKH Piebr-AKH	pELTFTSSWGa pELTFSSGWa	1008.4785 907.4308	This study; HMEL003881-PA
		*H. hecale*	Manse-AKH Piebr-AKH	pELTFTSSWGa pELTFSSGWa	1008.4785 907.4308	This study
		*Dryas iulia*	Manse-AKH Piebr-AKH	pELTFTSSWGa pELTFSSGWa	1008.4785 907.4308	This study
		*Acraea horta*	Manse-AKH Piebr-AKH Triin-AKH	pELTFTSSWGa pELTFSSGWa pELTFTPNWGa	1008.4785 907.4308 1045.5102	This study
		*Parthenos sylvia*	Manse-AKH Piebr-AKH	pELTFTSSWGa pELTFSSGWa	1008.4785 907.4308	This study
		*Bicyclus anynana*	Manse-AKH	pELTFTSSWGa	1008.4785	nBA.01-t08034-RA
		*Caligo memnon*	Manse-AKH	pELTFTSSWGa	1008.4785	This study
		*Dira clytus clytus*	Manse-AKH Dircl-AKH-I Dircl-AKH-II	pELTFTSSWGa pELTFSSGWGa pELTFSTGWa	1008.4785 964.4523 921.4465	This study
		*Danaus plexippus*	Manse-AKH Piebr-AKH	pELTFTSSWGa pELTFSSGWa	1008.4785 907.4308	This study; Köllisch et al. (2003)OWR54335.1 This study; OWR54334.1
		*D. chrysippus*	Manse-AKH	pELTFTSSWGa	1008.4785	This study
		*Greta oto*	Manse-AKH Piebr-AKH	pELTFTSSWGa pELTFSSWGa	1008.4785 907.4308	This study
		*Idea leuconoe*	Manse-AKH	pELTFTSSWGa	1008.4785	This study; OWR54335.1
		*Melitaea cinxia*	Manse-AKH Piebr-AKH	pELTFTSSWGa pELTFSSGWa	1008.4785 907.4308	This study; MCINX012977-PA This study
		*Aglais io*	Manse-AKH	pELTFTSSWGa	1008.4785	This study
		*A. urticae*	Manse-AKH	pELTFTSSWGa	1008.4785	This study; Köllisch et al., 2003
		*Vanessa atalanta*	Manse-AKH	pELTFTSSWGa	1008.4785	This study
		*V. cardui*	Manse-AKH	pELTFTSSWGa	1008.4785	Köllisch et al., 2000
		*Junonia (=Precis) coenia*	Manse-AKH	pELTFTSSWGa	1008.4785	Köllisch et al., 2003; JC_0008480-RA
Obtectomera, Gelechioidea	Stathmopodi-dae	*Atrijuglans hetaohei*	Manse-AKH Piebr-AKH	pELTFTSSWGa pELTFSSWGa	1008.4785 907.4308	AhAKH1 ID CL31877Contig1; Li et al., 2020 AhAKH2 ID CL18765Contig1; Li et al., 2020
Obtectomera, Pyraloidea	Pyralidae	*Galleria mellonella*	Manse-AKH	pELTFTSSWGa	1008.4785	XP_026761256.1
		*Plodia interpunctella*	Manse-AKH Chipa-AKH	pELTFTSSWGa pELTFSTGWGNa	1008.4785 1092.5109	Corzo et al., 2020
		*Amyelois transitella*	Chipa-AKH	pELTFSTGWGNa	1092.5109	XP_013191035.1
	Crambidae	*Chilo partellus*	Manse-AKH Chipa-AKH	pELTFTSSWGa pELTFSTGWGNa	1008.4785 1092.5109	This study
		*Chilo suppressalis*	Manse-AKH Chipa-AKH	pELTFTSSWGa pELTFSTGWGNa	1008.4785 1092.5109	ALM30296.1; Xu et al., 2016 ALM30297.1; Xu et al., 2016
		*Ostrinia furnacalis*	Manse-AKH NOVEL 2	pELTFTSSWGa pELTFSTGWGQa	1008.4785 1106.5265	XP_028164252 XP_028164238
Macroheterocera, Noctuoidea	Noctuidae	*Helicoverpa (=Heliothis) zea*	Manse-AKH Helze-HrTH	pELTFTSSWGa pELTFSSGWGNa	1008.4785 1078.4952	Jaffe et al., 1986; this study Jaffe et al., 1988; this study
		*Helicoverpa armigera*	Manse-AKH Helze-HrTH	pELTFTSSWGa pELTFSSGWGNa	1008.4785 1078.4952	AGH22544.1 AGH25545.1
		*Spodoptera frugiperda*	Manse-AKH Helze-HrTH	pELTFTSSWGa pELTFSSGWGNa	1008.4785 1078.4952	Köllisch et al., 2003; Gäde et al., 2008; Abdel-Latief and Hoffmann, 2007; this study
		*Spodoptera littoralis*	Manse-AKH Helze-HrTH	pELTFTSSWGa pELTFSSGWGNa	1008.4785 1078.4952	Gäde et al., 2008
		*Spodoptera exigua*	Manse-AKH Helze-HrTH	pELTFTSSWGa pELTFSSGWGNa	1008.4785 1078.4952	AXY04229.1; Llopis-Giménez et al., 2019 AXY04230.1; Llopis-Giménez et al., 2019
		*Lacanobia oleracea*	Manse-AKH Helze-HrTH Lacol-AKH	pELTFTSSWGa pELTFSSGWGNa pELTFTSSWGGa	1008.4785 1078.4952 1065.5000	Gäde et al., 2008
		*Mamestra brassicae*	Manse-AKH Helze-HrTH Lacol-AKH	pELTFTSSWGa pELTFSSWGGNa pELTFTSSWGGa	1008.4785 1078.4952 1065.5000	Fónagy et al., 2008; Gäde et al., 2008; Gäde et al. 2008 Gäde et al. 2008
		*Hadena bicruris*	Manse-AKH Helze-HrTH Lacol-AKH	pELTFTSSWGa pELTFSSGWGNa pELTFTSSWGGa	1008.4785 1078.4952 1065.5000	This study
		*Agrotis ipsilon*	Manse-AKH Helze-HrTH Lacol-AKH	pELTFTSSWGa pELTFSSGWGNa pELTFTSSWGGa	1008.4785 1078.4952 1065.5000	C0HL92; Interpretation of this study^‡^ Diesner et al., 2018 Diesner et al., 2018
		*Mythimna separata*	Lacol-AKH Helze-HrTH	pELTFTSSWGGa pELTFSSGWGNa	1065.5000 1078.4952	ALX27200.1 APJ36628.1
		*Trichoplusia ni*	Manse-AKH	pELTFTSSWGa	1008.4785	XP_026731718.1
	Erebidae	*Cyligramma latona*	Manse-AKH Helze-AKH	pELTFTSSWGa pELTFSSGWGNa	1008.4785 1078.4952	This study
		*Arctia plantaginis*	Manse-AKH Helze-HrTH	pELTFTSSWGa pELTFSSGWGNa	1008.4785 1078.4952	CAB3233079.1 CAB3224801.1
Macroheterocera, Bombycoidea	Saturniidae	*Actias luna*	Manse-AKH Manse-AKH-II	pELTFTSSWGa pELTFSSGWGQa	1008.4785 1092.5109	This study
		*Antherae yamamai*	Manse-AKH Antya-AKH	pELTFTSSWGa pELTFSPGWGQa	1008.4785 1102.5316	This study
	Bombycidae	*Bombyx mori*	Manse-AKH Bommo-AKH	pELTFTSSWGa pELTFTPGWGQa	1008.4785 1116.5473	Ishibashi et al., 1992; Gäde et al., 2008; NP_001104825.1 Gäde et al., 2008; NP_001124365.1
	Sphingidae	*Manduca sexta*	Manse-AKH Manse-AKH-II	pELTFTSSWGa pELTFSSGWGQa	1008.4785 1092.5109	Ziegler et al. (1985); Weaver et al. (2012) Weaver et al. (2012)
		*Deilephila elpenor*	Manse-AKH Manse-AKH-II	pELTFTSSWGa pELTFSSGWGQa	1008.4785 1092.5109	Weaver et al. (2012)
		*Laothoe populi*	Manse-AKH Manse-AKH-II	pELTFTSSWGa pELTFSSGWGQa	1008.4785 1092.5109	Weaver et al. (2012)
		*Smerinthus ocellata*	Manse-AKH	pELTFTSSWGa	1008.4785	Weaver et al. (2012)
		*Acherontia atropos*	Manse-AKH Manse-AKH-II	pELTFTSSWGa pELTFSSGWGQa	1008.4785 1092.5109	Weaver et al. (2012)
		*Hyles lineata*	Manse-AKH Manse-AKH-II	pELTFTSSWGa pELTFSSGWGQa	1008.4785 1092.5109	This study
		*Agrius convolvuli*	Manse-AKH Manse-AKH-II	pELTFTSSWGa pELTFSSGWGQa	1008.4785 1092.5109	This study
		*Sphinx ligustri*	Manse-AKH Manse-AKH-II	pELTFTSSWGa pELTFSSGWGQa	1008.4785 1092.5109	This study
		*Daphnis nerii*	Manse-AKH Manse-AKH-II	pELTFTSSWGa pELTFSSGWGQa	1008.4785 1092.5109	This study
		*Hippotion eson*	Manse-AKH-I Manse-AKH-II Hipes-AKH-I Hipes-AKH-II Hipes-AKH-III	pELTFTSSWGa pELTFSSGWGQa pELTFTSSWa pELTFTSTWa pELTFTSTWGa	1008.4785 1092.5109 951.4571 965.4727 1022.4942	Gäde et al., 2013
		*Hippotion celerio*	Manse-AKH Manse-AKH-II Hipes-AKH-I Hipes-AKH-II Hipes-AKH-III	pELTFTSSWGa pELTFSSGWGQa pELTFTSSWa pELTFTSTWa pELTFTSTWGa	1008.4785 1092.5109 951.4571 965.4727 1022.4942	Gäde et al., 2013

The phylogenetic outline is as per Mitter et al. (2017) and Kawahara et al. (2019). *Mature peptide sequence: in the case of sequences derived from nucleotide databases, the post-translational modifications (i.e., blocked termini) are deduced from the characteristic features of previously sequenced AKHs. Note that the N-terminal pyroglutamic acid (pE) can arise from the cyclization of a glutamine (Q) or, more rarely, from a glutamate (E) residue. In the case of prepro-AKH sequences translated from mRNA, all have a glutamine amino acid residue at position 1 of the uncleaved AKH sequence. Novel peptide sequences deduced from data mining are consecutively numbered (1–2) – note that we have not confirmed the data experimentally. **Published work or database accession number. ^#^Vanca-AKH is not considered a mature AKH peptide but an incompletely processed form of Manse-AKH. Although it occurs in many other Lepidoptera species, it is only indicated here. ^‡^Diesner et al. (2018) overlooked Manse-AKH in their interpretations of putative preprohormones identified from an *A. ipsilon* transcriptome and grouped the Manse-AKH precursor together with the Lacol-AKH precursor as “adipokinetic 1” [see data in Table 2, as well as mass spectrometry data in Figure 4B of Diesner et al. (2018), where the (M + Na)^+^ adduct corresponding to Manse-AKH is shown].

Next, a decapeptide that had some adipokinetic and pronounced trehalose-elevating activity in the cornear moth *Heliothis* (=*Helicoverpa*) *zea* (family: Noctuidae) was sequenced from the CC of this species and was, hence called a hypertrehalosemic hormone (Helze-HrTH, pELTFSSGWGN amide; Jaffe et al., 1988). It has since been found in other noctuids as well (see Table 1). In the common commercially exploited silk moth *B. mori* (family: Bombycidae) genomic and physiological information, as well as mass spectrometric measurements led to the identification of another decapeptide (Bommo-AKH: pELTFTPGWGQ amide) with lipid-mobilizing activity besides Manse-AKH (Liebrich and Gäde, 1995; Gäde et al., 2008; Roller et al., 2008). In the same year it was found by mass spectrometry that the CC of two owlet moths (family: Noctuidae), the bright-line brown-eye moth *Lacanobia oleracea* and the cabbage moth *Mamestra brassicae*, synthesized a third AKH peptide (besides Manse-AKH and Helze-HrTH); this was a decapeptide (Lacol-AKH, pELTFTSSWGG amide) which is near-identical to Manse-AKH save for an extra glycine residue (Gäde et al., 2008). A unique case of five different AKHs in the CC occurs in two species of the genus *Hippotion* (family: Sphingidae) – mass spectrometry unraveled the existence of three novel members of the AKH family: the octapeptides Hipes-AKH-I (pELTFTSSWamide) and Hipes-AKH-II (pELTFTSTW amide), as well as the nonapeptide Hipes-AKH-III (pELTFTFSTWG amide); Manse-AKH and Manse-AKH-II were also present (Gäde et al., 2013). All five peptides mobilize lipids after injection into *Hippotion eson* (Gäde et al., 2013), and quantitative studies showed that lipids are the main fuel during a 15 min flight of the sphingid moth, with only a small contribution by trehalose oxidation (Liebrich and Gäde, 1995). The latest addition to Lepidopteran AKHs is from the CC of the large cabbage white butterfly *Pieris brassicae* (family: Pieridae) where a lipid-mobilizing peptide was mass spectrometrically sequenced as an octapeptide (Piebr-AKH, pELTFSSGW amide) which co-occurs with Manse-AKH and a biologically inactive, non-amidated peptide that resembles Manse-AKH and is code-named Vanca-AKH (Marco and Gäde, 2017). Vanca-AKH was first elucidated from the nymphalid painted lady butterfly, *Vanessa cardui* (pELTFTSSWGGK; Köllisch et al., 2000) and has since been identified in other Lepidoptera (Table 1 shows Vanca-AKH only in the earliest known appearance, viz. in *Papilio demodocus*, family Papilionidae). Although Vanca-AKH had biological activity under certain conditions in *V. cardui* (Köllisch et al., 2000), it is not active in unmanipulated *P. brassicae* which reacted well to Manse-AKH and Piebr-AKH (Marco and Gäde, 2017), hence, Vanca-AKH is rather viewed as an incompletely processed peptide from the AKH-precursor. Such processing “mistakes”/intermediates are known from AKHs of other orders (Diptera, Coleoptera) as well (see, for example, Predel et al., 2004; Gäde and Marco, 2011).

It is evident from the above that, although there are about 160,000 extant species of Lepidoptera described, the number of investigated species with respect to AKH is small. Interestingly, all nine AKHs known so far by (bio)chemical sequencing methods are unique for this order, i.e., have not been found to be synthesized in the CC of any other insect order. This makes this group of peptides in Lepidoptera very interesting for further research to look for an order-specific control agent. The effort to find species-specific, biorational and biostable control agents (peptide mimetics that are based on the insect’s own hormones), so-called “green” insecticides, is high on the agenda of basic research ventures to target and control pest insects of agriculture, horticulture and forestry, with little or no negative effect on beneficial insects, other organisms and the environment. So, for example, head-to-tail cyclic and other restricted conformation analogs of insect neuropeptides, such as AKH and diapause hormone, have been synthesized and tested in bioassays to study their active conformations and target selectivity (Zhang et al., 2011; Abdulganiyyu et al., 2020a). Amongst the diapause hormone mimetics both agonists and antagonists are identified (Zhang et al., 2011), while a cyclic AKH mimetic based on a locust peptide (Locmi-AKH-I) demonstrates selectivity in *in vitro* receptor assays, activating the AKH receptor of the desert locust but failing to activate the AKH receptor of the honeybee even at pharmacological concentrations (Abdulganiyyu et al., 2020a). Molecular dynamic analyses indicated that the cyclic mimetic failed to enter the binding pocket of the honeybee receptor 3D model during docking simulations (Abdulganiyyu et al., 2020a).

Whilst the above-mentioned mimetics are not yet in the field trial stage, such studies and the current investigation provide the groundwork for future “green” insecticides for, not surprisingly, several lepidopteran species also represent those insects that are a real threat to human food security, and with the general rise in resistance to chemical pesticides, the need for “green” insecticides as part of an integrated pest management strategy is becoming more urgent than ever. Before much effort and funds are invested in such a research area to make peptide mimetics based on a lepidopteran AKH, for example, one should first broaden the information base on the complement of AKHs in Lepidoptera.

The current study, therefore, had several objectives and is not hypothesis-driven:

1. To investigate more members of the order Lepidoptera and determine the structure of the respective AKH. Not only species from different superfamilies and families were analyzed but also from various regions of the world.
2. We are interested in the physiological function of AKHs and, therefore, checked for a few selected species whether the AKH is involved in lipid and/or carbohydrate regulation. In addition, we used *P. brassicae*, a recent introduced pest species in South Africa, as test case to ascertain whether all the known lepidopteran AKHs could successfully trigger the adipokinetic signaling system in a pest species. This information could be useful for further investigations into a possible order-specific control agent (lepidopteran-specific “green” insecticide).
3. As we had previously successfully implemented the use of the primary structure of AKHs in a few insect orders to verify certain phylogenetic trends and ancestral relationships (Gäde, 1989; Gäde and Marco, 2005, 2017; Gäde et al., 2020), we also wanted to use the structural information of the investigated AKHs to trace the general phylogeny of Lepidoptera and sketch a possible molecular evolution of the AKHs in this order.

## Materials and Methods

### Insects

Corpora cardiaca were dissected from female and/or male adults. Specimens were either caught in the field by netting, were purchased from breeders or were received as a gift from a research institution or a commercial company. Some species were bred from eggs (*Actias luna*, *Catopsilia florella*, *P. brassicae*, and *Sphinx ligustri*). In total, 34 species of Lepidoptera were studied for their AKH complement; details of the species and the taxonomic affiliations are given below. For the latter, the phylogenetic outline given by Mitter et al. (2017) and Kawahara et al. (2019) were followed as explained in section “Introduction.” Figure 1 supplies a quick orientation to a much-simplified phylogenetic tree, showing the superfamilies investigated in the current study.

#### Superfamily Tortricoidea

Three species of leaf rollers (family: Tortricidae) were investigated. Pupae of the codling moth (*C. pomonella*; 20 CCs prepared), the litchi moth (*Cryptophlebia peltastica*; 20 CCs prepared) and the false codling moth [*Thaumatotibia* (=*Cryptophlebia*) *leucotreta*; 20 CCs prepared] were a gift of the River Bioscience Group (Port Elizabeth, South Africa). Larvae of these species are major pests to agricultural crops such as apples, pears and litchis.

#### Superfamily Papilionoidea

Nineteen species of butterflies were investigated. Two species belong to the swallowtails (family: Papillionidae): *P. demodocus* (4 CCs prepared) were collected by netting in a private garden in Cape Town in the austral summer around a citrus tree, *Papilio memnon* (8 CCs prepared) was purchased as pupae from a commercial breeder in the United Kingdom.

Two species were of the whites (family: Pieridae): eggs of the African migrant (*C. florella*; 7 CCs prepared) were collected in a private garden in Cape Town from leaves of the monkey pod (*Cassia* ssp.) in March and reared to adult eclosion fed with those leaves. Adults of the brimstone (*Gonepteryx rhamni*; 4 CCs prepared) were caught by netting in a private garden in Bad Iburg (Germany) in April.

Fifteen species of the brush foots (family: Nymphalidae) from various subfamilies were investigated: pupae of the heliconid red lacewing (*Cethosia biblis*; 1 CC prepared), the postman (*Heliconius melpomene*; 6 CCs prepared), the tiger longwing (*Heliconius hecale*; 3 CCs prepared), the clipper (*Parthenos sylvia*; 5 CCs prepared) and the monarch butterfly (*Danaus plexippus*; 4 CCs prepared) came from a commercial breeder from the United Kingdom; adults of the giant owl (*Caligo memnon*; 2 CCs prepared), the glasswing butterfly (*Greta oto*; 10 CCs prepared), the Julia butterfly (*Dryas iulia*; 15 CCs prepared) and the paperkite butterfly (*Idea leuconoe*; 8 CCs prepared) were a gift from Butterfly World (Klapmuts, South Africa), CCs of the Glanville fritillary (*M. cinxia*; 67 CCs) were a gift from H. Fescemyer (The Pennsylvania State University, United States); adults of the Red Admiral (*Vanessa atalanta*; 4 CCs prepared), the European peacock (*Aglais io*; 4 CCs prepared) and the small tortoiseshell (*Aglais urticae*; 2 CCs prepared) were collected by netting in a private garden in Bad Iburg (Germany) on flowering *Buddleia*; adults of the garden Acraea (*Acraea horta*; 15 CCs prepared), the African Monarch (*Danaus chrysippus*; 3 CCs prepared) and the Cape autumn widow (*Dira clytus clytus*; 15 CCs prepared) were collected by netting in a private garden or at the grounds of the University of Cape Town in the austral summer.

#### Superfamily Pyraloidea

One species was investigated: pupae of the spotted stemborer (*Chilo partellus*; 20 CCs prepared) were a gift of Frank Chidawanyika (University of Bloemfontein, South Africa) and came originally from a culture held at the International Centre for Insect Physiology and Ecology (ICIPE) in Kenya. This species is especially damaging to staple food plants of maize and sorghum in eastern and southern Africa (Khadioli et al., 2014).

#### Superfamily Noctuoidea

Four species were investigated. CCs from adults of fall armyworm moths (*S. frugiperda*; 20 CC) and corn earworm moths (*H. zea*; 40 CC) were a gift from Howard W. Fescemyer (The Pennsylvania State University, United States), pupae of the Lychnis moth (*Hadena bicruris*; 11 CCs prepared) were a gift of Carmen Villacañas de Castro (University of Bremen, Germany) and adults of the cream striped owl moth (*Cyligramma latona*; 5 CCs prepared) were collected by netting at the grounds of the University of Namibia. Armyworm and earworm larvae have serious pest status with respect to a variety of food plants (Cunningham and Zalucki, 2014), while *H. bicruris* is a specialist nocturnal nursery pollinator of *Silene latifolia* (=*Melandrium album*) (Brantjes, 1976).

#### Superfamily Bombycoidea

Six species were investigated. The two species from the family Saturniidae were the luna moth (*Actias luna*; 7 CCs prepared) which were reared from eggs on walnut leaves (*Juglans regia*), and the Japanese oak silk moth (*Antheraea yamamai*; 4 CCs prepared) of which pupae were received as gift from Thomas Olthoff (University of Hamburg). Four species from the family Sphingidae were studied: the privet hawk moth (*S. ligustri*; 4 CCs prepared) which were reared from eggs on privet (*Ligustrum vulgare*), the Oleander hawkmoth (*Daphnis nerii*; 2 CCs prepared) of which the adults were collected in October near Heraklion (Greece) on a private property site, the convolvulus hawkmoth (*Agrius convolvuli*; 1 CC prepared) of which one adult was fortuitously netted in Windhoek (Namibia) and the white-lined sphinx moth (*Hyles lineata*; 4 CCs prepared) whose pupae were received as gift from Martin von Arx (University of Arizona, United States).

### Biological Assays

Bioassays were performed with only a few species when adult specimens were available in sufficient numbers. *H. eson* and *P. brassicae* specimens were reared from eggs which were collected in a private garden in Cape Town, as described previously (Marco and Gäde, 2017, 2019); adults of both genders were used on the first or second day after eclosion. Individuals of *A. horta* used in bioassays were netted in a private garden in Cape Town; animals of unspecified age and both genders were used on the day of collection. Bioassay acceptor insects were kept individually under an up-ended funnel on moist tissue paper at ambient temperature (22.5 ± 1°C) in the laboratory for about 2 h for acclimatization and keeping them at rest. A first hemolymph sample of 0.5 μl was taken laterally from the metathorax or abdomen, or directly from the dorsal heart in the centerline of the abdomen, with a disposable glass microcapillary (Hirschmann Laborgeräte, Eberstadt, Germany), blown into a test tube of concentrated sulfuric assay and the animal was then injected ventrolaterally into the abdomen with 2 or 3 μl of either distilled water, a crude CC extract, or a synthetic peptide delivered in distilled water via a Hamilton fine-bore 10 μl syringe. After injection, the animal was returned to rest for 90 min after which a second hemolymph sample was taken from the same individual. The hemolymph samples in sulfuric acid were then measured for vanillin-positive material (=total lipids) or anthrone-positive material (=total carbohydrates) according to the phosphovanillin method (Zöllner and Kirsch, 1962) and anthrone method (Spik and Montreuil, 1964), respectively, as modified by Holwerda et al. (1977).

Student’s paired *t*-test was used to compare the concentration of metabolites (lipids or carbohydrates) in the hemolymph before and after the injection of a test solution. Differences were considered significant at *p* < 0.05.

### Dissection of Corpora Cardiaca, Peptide Extraction, and Separation, Mass Spectrometry and Sequence Analysis

The only source of AKH, the corpora cardiaca, were dissected from the heads of adult specimens of each of the 34 lepidopteran species under investigation with the aid of a stereomicroscope at 20 to 40-fold magnification. The neuroendocrine glands were placed into a microcentrifuge tube containing 80% v/v methanol, extracted as described previously (Gäde et al., 1984), and dried in a vacuum-centrifuge. Thereafter, extracts were dissolved in either distilled water for use in biological assays, or in 50 μl of aqueous formic acid for liquid chromatography tandem positive ion electrospray mass spectrometry (LC-ESI) on an LTQ XL linear ion trap instrument (Thermo Fisher Scientific, San Jose, CA, United States), as previously outlined in detail (Kodrík et al., 2010).

Exact mass and elemental composition were acquired by LC-ESI high-resolution mass spectrometry (HRMS) using the same Jupiter RP Proteo column and gradient elution, but a HRMS Orbitrap Q-Exactive Plus mass spectrometer (Thermo Fisher Scientific) equipped with a HESI-II ion source. Positive ESI mass spectra were scanned every 2.1 s and were acquired at resolution *R* = 70,000 with an internal lock mass *m/z* 622.02896 of hexakis(2,2-difluoroethoxy)phosphazene using the mass range of 450–1250 Da.

### Synthetic Peptides

Synthetic peptides (see Table 1 for full name and sequence) were used in biological assays and to confirm interpretations of mass spectrometric data. Peptides with the code-names Manse-AKH, Helze-AKH, Lacol-AKH and Peram-CAH-II were synthesized by Pepmic Co., Ltd. (Suzou, China); Bommo-AKH, Manse-AKH-II, Piebr-AKH, Hipes-AKH-I, -II, and -III were custom-synthesized by Kevin Clark (Department of Entomology, University of Georgia, Athens, Georgia), while the novel peptides of this study (Dircl-AKH-I and -II, Chipa-AKH, and Antya-AKH) were also made by Pepmic Co., Ltd.

### Mining of AKH Sequences From Publicly Available Databases

We investigated AKH sequences from 34 lepidopteran species via MS in the current study. To gain greater insight into the distribution of AKHs in this speciose order we further tried to extend the knowledge base by using bioinformatic searches to identify Lepidoptera AKHs from protein, genomic and/or EST databases. Triant et al. (2018) provides a comprehensive list of Lepidoptera genomes and relevant databases. Some AKH sequences were retrieved directly from the 1KITE initiative (http://www.1kite.org/) and from Lepbase,^2^ a useful hub for lepidopteran genomes that includes new genome assemblies and annotations (Challis et al., 2016). AKH sequences were accessed using BLAST (Basic Local Alignment Search Tool) search function with the nucleotide sequence of Manse-AKH or Bommo-AKH as query. Sequences were also retrieved via the National Center for Biotechnology Information^3^.

## Results and Discussion

### Presence of AKH Peptides in Select Lepidoptera Species and Physiological Action

Biological assays were performed with a few lepidopteran species to screen for hyperlipemic (and sometimes also) hypertrehalosemic activity embedded in the tested CC. Different permutations of a well-established *in vivo* biological assay system were used: (i) CC extracts were injected into adult *H. eson* – a well-characterized sphingid moth species with respect to metabolic assays and AKHs that elicit a hyperlipemic response (Gäde et al., 2013); (ii) CC extracts were injected into adult *A. horta* – a nymphalid butterfly species that is investigated here for the first time with respect to metabolic activity; and (iii) synthetic peptides of all biochemically known lepidopteran AKHs were injected into adult *P. brassicae* – a butterfly species that responds to AKHs with hyperlipemia (see Marco and Gäde, 2017); the latter dataset is presented and discussed below (see section “Adipokinetic Response of Lepidoptera AKH Family Bioanalogs: Potential for “Green” Insecticides?”). These acceptor species were selected based on availability of adult specimens in high enough numbers to provide statistically relevant data. From previously gained knowledge, *H. eson* and *P. brassicae* were only tested for an adipokinetic response, whereas *A. horta* (hitherto untested in metabolic assays) was investigated for an adipokinetic, as well as a hypertrehalosemic response. Table 2A shows that the circulating level of carbohydrates are much higher (more than double) in resting *A. horta* specimens than the lipid concentration, which we interpret as an indicator that trehalose is the preferred metabolic fuel of the garden *Acraea*. Interestingly, when half a pair of CC from *A. horta* was conspecifically injected, *A. horta* responded with a significant rise in the lipid concentration compared to water-injected animals, as well as a significant, pronounced increase in the carbohydrate concentration in the hemolymph (Table 2A). This butterfly, thus, utilizes both metabolites, and this was unequivocally corroborated following injection of AKH peptides that are identified as endogenous to the CC of *A. horta* (see section “Mass Spectrometric-Derived Results” below), viz. Manse-AKH, Piebr-AKH, and Triin-AKH: each peptide triggered the signal transduction cascade in *A. horta* to increase both the lipid and the carbohydrate concentration in the hemolymph – although the hypertrehalosemic effect is stronger than the adipokinetic effect, both metabolites are mobilized from the fat body in response to the respective endogenous AKH peptides when injected in synthetic form at a low concentration (Table 2A). Injection of half a pair of CC from the autumn brown butterfly, *Dira clytus clytus*, into *A. horta* significantly increased the carbohydrate concentration compared to water-injected controls (Table 2A) which indicated the presence of an AKH peptide in the CC of autumn brown butterfly that interacted with the AKH receptor of *A. horta* to turn on the signal transduction cascade pathway. Due to insufficient numbers of *D. clytus clytus* we could not perform conspecific assays and did not have enough crude CC extract to test their lipid mobilizing effect in *A. horta*.

**TABLE 2:** Bioassays for adipokinetic and hypertrehalosemic activity of crude methanolic extracts of butterfly corpora cardiaca (*Acraea horta*, *Dira clytus clytus*, and *Cyligramma latona*, and of synthetic peptides in two acceptor species: *A. horta* (butterfly) and *Hippotion eson* (moth).

**Treatment**	**Hemolymph lipids (mg ml ^–1^)**					**Hemolymph carbohydrates (mg ml ^–1^)**				
	***n***	**0 min**	**90 min**	**Difference**	P*	***n***	**0 min**	**90 min**	**Difference**	P*
**A. Acceptor insect: *A. horta***										
Control (10 μl distilled water)	15	13.51 ± 4.37	13.21 ± 4.33	−0.30 ± 1.94	NS	13	49.60 ± 8.87	50.03 ± 8.91	0.43 ± 2.94	NS
*A. horta CC* extract (0.5 gland pair equivalent)	14	12.87 ± 6.36	16.93 ± 7.37	4.06 ± 2.55	0.00002	6	46.57 ± 1.26	51.57 ± 2.30	5.00 ± 2.04	0.0009
*D. clytus clytus CC* extract (0.5 gland pair equivalent)		**Not tested**				7	42.60 ± 5.90	54.45 ± 8.70	11.85 ± 4.02	0.00012
Manse-AKH (10 pmol)	6	13.41 ± 5.32	20.01 ± 4.72	6.60 ± 2.93	0.00002	6	50.96 ± 10.22	61.51 ± 9.99	10.55 ± 3.33	0.00029
Piebr-AKH (10 pmol)	10	10.13 ± 3.87	16.35 ± 5.18	6.21 ± 2.99	0.001	5	44.19 ± 3.64	53.06 ± 6.91	8.87 ± 4.01	0.0039
Triin-AKH (10 pmol)	6	15.98 ± 4.64	23.72 ± 6.31	7.74 ± 2.85	0.00058	6	30.59 ± 5.73	42.43 ± 14.97	11.84 ± 12.67	0.036
**B. Acceptor insect: *H. eson***							**Not tested**			
Control (5 μl distilled water)	6	44.59 ± 10.21	47.61 ± 11.55	3.01 ± 9.1	NS					
*C. latona CC* extract (0.5 gland pair equivalent)	7	31.23 ± 10.57	59.50 ± 15.68	28.27 ± 13.67	0.001					
*A. horta CC* extract (0.5 gland pair equivalent)	5	38.78 ± 8.84	58.29 ± 17.84	19.51 ± 10.23	0.0065					
Lacol-AKH (10 pmol)	7	28.70 ± 21.23	57.27 ± 29.13	28.57 ± 9.15	0.00008					

Data are presented as Mean ± SD. *Paired *t*-test was used to calculate the significance between pre- and post-injection. NS, not significant.

In heterologous bioassays with the sphingid moth *H. eson*, the injection of half a pair of CC from *A. horta* and of the noctuid moth *C. latona* resulted in a clear adipokinetic response when compared to the water-injected control group and was as high as or about 70% of the response to the decapeptide Lacol-AKH (Table 2B), which had previously been shown to have a full effect in *H. eson* (Marco and Gäde, 2015).

What can we conclude from these physiological experiments? The CC of all investigated species here synthesize one or more endogenous peptide that belongs to the AKH peptide family. In the case of heterologous injections, a positive adipokinetic or hypertrehalosemic response means that either the donor species contains exactly the same peptide(s) as the acceptor species or that the AKH receptor of the acceptor species recognizes a slightly modified peptide – this is not unusual for AKH receptors as, for example, structure activity studies with *H. eson* show (Marco and Gäde, 2019). In the case of *A. horta* this species has higher concentrations of carbohydrates than lipids in its hemolymph at rest which may be a hint that metabolism is carbohydrate-based since a correlation between the most abundant fuel substrate in an insect and the preferred utilization of a fuel was previously shown in insects (Gäde et al., 2004). Second, mobilization of stored fuels by a CC extract clearly pointed to a preference of carbohydrates versus lipids. Third, this hypertrehalosemic result could also be evoked by relatively low doses of all three synthetic AKHs that we find to be present in the CC of *A. horta* (see section “Mass Spectrometric-Derived Results” below). These results with *A. horta* are reminiscent of findings with the noctuid moth *H. zea*, where the endogenous decapeptide Helze-HrTH elicited an increase of both hemolymph metabolites, lipids and carbohydrates, but the hypertrehalosemic response was much more pronounced (Jaffe et al., 1988). In the sphingid moth *M. sexta*, the endogenous nonapeptide Manse-AKH is also responsible for the control of carbohydrate and lipid metabolism but this is stage-specific: in larvae the carbohydrate metabolic pathway is affected and in adults the lipids (Ziegler et al., 1990). Although not as rigorously investigated as in *M. sexta*, the bombycid *B. mori* appears to have the same type of regulation: in larvae AKH is involved in the homeostasis of the trehalose concentration in the hemolymph (Oda et al., 2000), whereas in adults CC injection and simulated flight increase hemolymph lipids (Liebrich and Gäde, 1995). For most other investigated lepidopteran species, it is only known that adults react with hyperlipemia (see, for example, Gäde et al., 2013; Marco and Gäde, 2017). The current results have shown quite clearly that AKH is involved in metabolic regulation, but it needs to be determined at a case to case event whether the endogenous AKH influences carbohydrate or lipid metabolism or both. Is there a link between flight distance and the metabolite fueling the intense aerobic activity? Lipids in the form of triglycerides are far less bulky to store in the fat body compared with glycogen, and triglycerides have a higher caloric content per unit of weight than glycogen, hence, it comes as no surprise that lipid metabolism is prevalent in lepidopterans that engage in migratory (long distance) flights, such as sphingid moths (Liebrich and Gäde, 1995; Ziegler and Schulz, 1986a,b), cabbage moth and -butterfly (Fónagy et al., 2008; Marco and Gäde, 2017) and the nymphalid painted lady (Köllisch et al., 2000). In *Helicoverpa* moths, such as *H. zea* that practices short-range, long-range, and migratory movements,^4^ both lipids and carbohydrates are mobilized (Jaffe et al., 1988). *A. horta* engages only in very short, flitting flights in the garden from one nectar source to the next, and this would fit with a mainly carbohydrate-based metabolism.

### Isolation and Mass Spectral Analyses of Adipokinetic Hormones From Various Lepidoptera Species

Separation, detection, sequence assignment, and confirmation of AKHs from Lepidoptera species were carried out with mass spectrometry, as described below. Following on from this, we show and discuss the outcome of our search for AKH structures in all investigated lepidopteran species, including those where transcriptomic/genomic mining resulted in knowledge of AKH sequences.

#### Mass Spectrometric-Derived Results

##### Tortricoidea: *Cydia pomonella*

**FIGURE 2:**
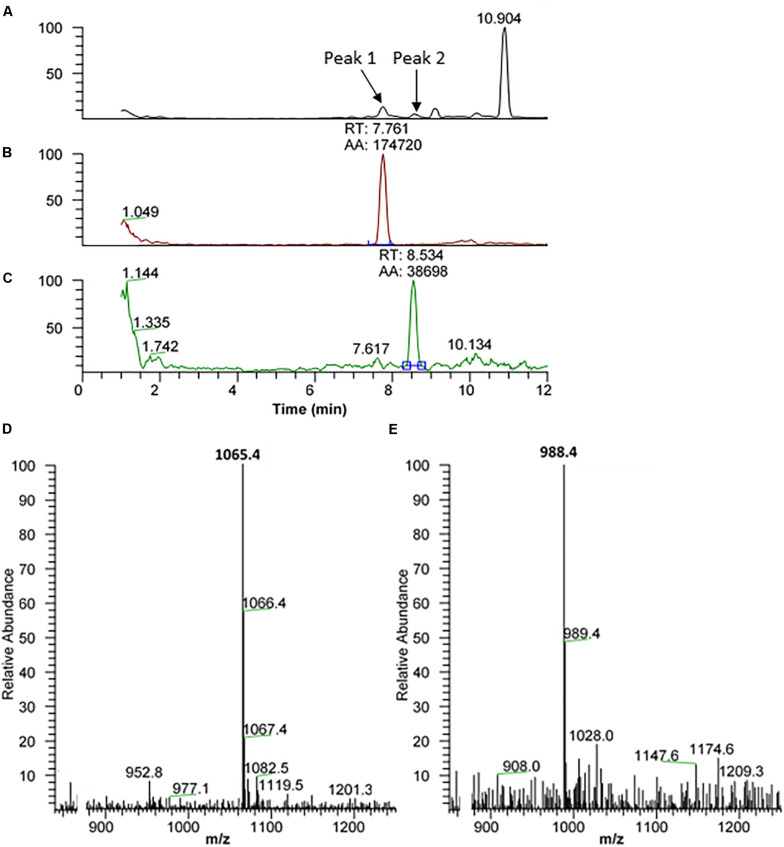
Liquid chromatographic (LC) positive electrospray ionization (+ESI) mass spectrometric (MS) analysis of a methanolic extract from the corpus cardiacum of the codling moth *Cydia pomonella*. **(A)** Base peak chromatogram obtained by LC-MS analysis showing detection of two AKH peptides labeled peak 1 and peak 2 at 7.76 and 8.53 min, respectively. **(B)** The extracted LC-MS chromatogram of peak 1 at 7.76 min with (M + H)^+^ at *m*/*z* 1065.5 (see **D**). **(C)** The extracted LC-MS chromatogram of peak 2 at 8.53 min with (M + H)^+^ at *m/z* 988.4 (see **E**).

**FIGURE 3:**
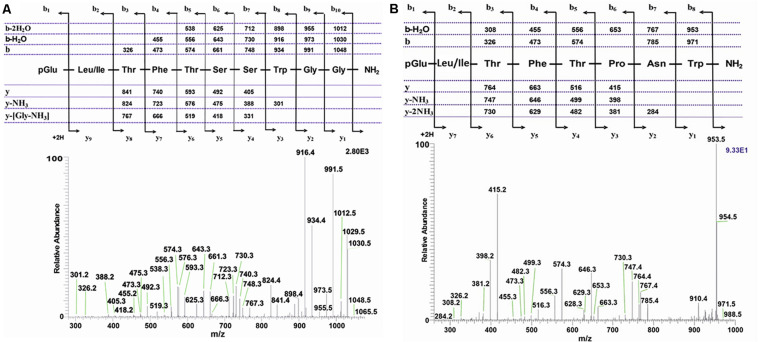
Collision-induced dissociation (CID) tandem MS + ESI spectra. **(A)** The CID spectrum of the ion (M + H)^+^ at *m*/*z* 1065.5 in Figures 2B,D from *C. pomonella*. **(B)** The CID spectrum of the ion (M + H)^+^ at *m*/*z* 988.4 in Figures 2C,E from *C. pomonella*. The insets show the proposed peptide sequences together with diagnostic fragment ions observed in the MS^2^ spectra.

The methanolic CC extract of the codling moth *C. pomonella* was fractionated by reversed-phase liquid chromatography (LC), and the peptides detected by positive ion electrospray mass spectrometry (+ESI-MS). Figure 2A shows the base peak chromatogram, whereas Figures 2B,C depict extracted mass peaks of AKHs at 7.76 and 8.53 min, respectively, with the corresponding (M + H)^+^ mass ions at *m/z* 1065.4 (Figure 2D) and *m/z* 988.4 (Figure 2E). The primary structure of these peak materials was deduced from the tandem MS^2^ spectra obtained by collision-induced dissociation (CID) of the respective *m/z* ions. The spectrum of *m/z* 1065.5 (Figure 3A) with clearly defined b, y, b-H_2_O, y-NH_3_, and other product ions allowed an almost complete assignment of a typical decapeptide member of the AKH family under the assumption that the peptide has a characteristic pyroglutamate residue at the N-terminus (see schematic inset in Figure 3A). All other amino acids are assigned except at position two where the remaining mass of 113 can be accredited to one of the isomers: leucine or isoleucine. A peptide with the sequence pGlu-Leu-Thr-Phe-Thr-Ser-Ser-Trp-Gly-Gly amide, thus with the Leu^2^ isomer, had previously been sequenced in certain noctuid moths and is called Lacol-AKH (see section “Introduction”). The second CID spectrum of the peptide at *m/z* 988.4 (Figure 3B) led to the interpretation of an octapeptide member of the AKH family with the sequence pGlu-Leu/Ile-Thr-Phe-Thr-Pro-Asn-Trp amide which, with Leu^2^, is well-known under the name Peram-CAH-II as one of the two peptides found in the CC of blattid cockroaches (see Gäde, 2009). Since we had previously established that isobaric Leu^2^/Ile^2^ containing peptides have different LC retention times (Gäde et al., 2003, 2016) and since we had the synthetic peptides Lacol-AKH and Peram-CAH-II available, we could prove via co-elution experiments (Supplementary Figures 1A–C) that both assigned peptides of *C. pomonella* have Leu and not Ile as the second amino acid residue in the amino acid sequence and are, indeed, the peptides known as Lacol-AKH and Peram-CAH-II.

##### Papilionoidea: *Dira clytus clytus*

**FIGURE 4:**
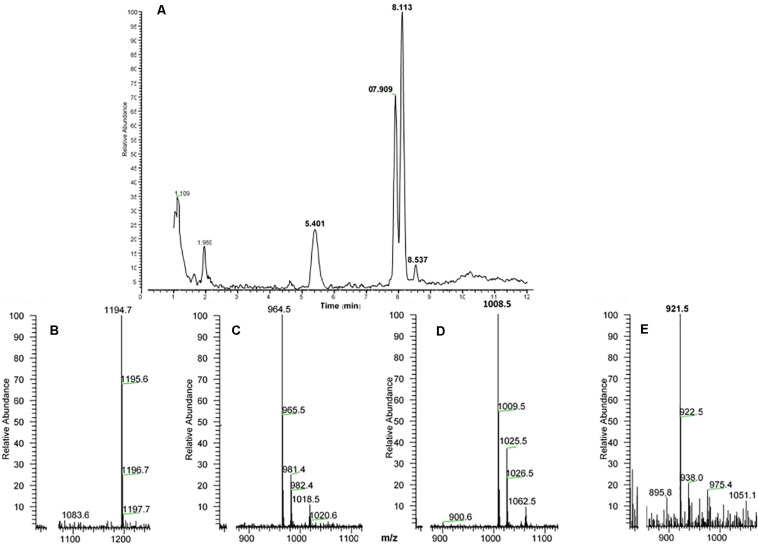
LC-MS analysis of a methanolic extract from the corpus cardiacum of the Cape autumn widow *Dira clytus clytus*. **(A)** Base peak chromatogram obtained by LC-MS analysis showing detection of four AKH peptides labeled by retention times (RT) at 5.40, 7.91, 8.11, and 8.53 min, respectively. The background subtracted + ESI mass spectra are shown for **(B)** the ion at RT of 5.40 min and at *m*/*z* 1194.7; **(C)** the ion at RT 7.91 and at *m*/*z* 964.5; **(D)** the ion at RT of 8.11 and at *m*/*z* 1008.5; **(E)** the ion at RT of 8.53 and at *m*/*z* 921.5.

**FIGURE 5:**
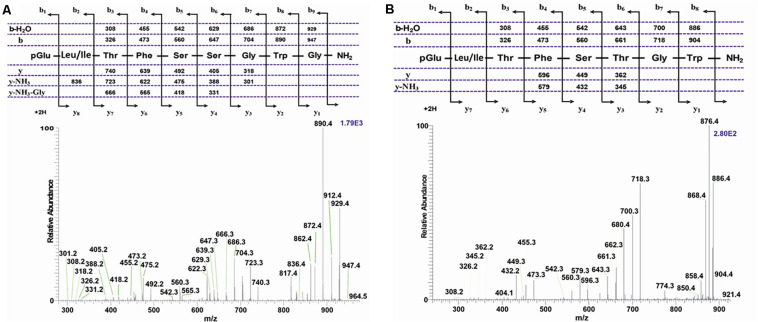
CID tandem MS + ESI spectra. **(A)** The CID spectrum of the ion (M + H)^+^ at *m*/*z* 964.5 in Figure 4C from *D. clytus clytus*. **(B)** The CID spectrum of the ion (M + H)^+^ at *m*/*z* 921.5 in Figure 4E from *D. clytus clytus*. The insets show the proposed peptide sequences together with diagnostic fragment ions observed in the MS^2^ spectra.

An extract from the CC of the Cape autumn widow *D. clytus clytus* gave information in LC-MS of four possible AKHs (Figure 4A): the first eluting peak with retention time of 5.40 min, displayed *m/z* 1194.7 (Figure 4B) with a primary sequence of pGlu-Leu-Thr-Phe-Thr-Ser-Ser-Trp-Gly-Gly-Lys-OH as interpreted from CID measurements (Supplementary Figure 2A). This amino acid sequence was shown to occur in the painted lady *V. cardui* and other nymphalids (see section “Introduction”) and is the incompletely processed AKH known as Vanca-AKH which has biological activity only under certain (unnatural) bioassay conditions (Köllisch et al., 2000; Marco and Gäde, 2017). The other three peaks at retention times 7.91, 8.11, and 8.53 min (Figure 4A) each correspond to a mature AKH with a respective *m/z* of 964.5 (Figure 4C), 1008.5 (Figure 4D) and 921.5 (Figure 4E). CID (Supplementary Figure 2B) and co-elution experiments (Supplementary Figures 2C–E) proved that the peptide with (M + H)^+^ 1008.5 is a nonapeptide with the sequence pGlu-Leu-Thr-Phe-Thr-Ser-Ser-Trp-Gly amide, well-known as Manse-AKH and found in *M. sexta* and a number of other butterflies and moths (see section “Introduction”). The other two peptides are novel and have not previously been found in any insect species. CID (Figure 5A) and co-elution (Supplementary Figures 2F–H) assigned the sequence for the peptide at *m/z* 964.5 as another nonapeptide: pGlu-Leu-Thr-Phe-Ser-Ser-Gly-Trp-Gly amide. We name it Dircl-AKH-I; it is identical to the first nine amino acid residues of Manse-AKH-II which is a decapeptide with a 10th residue, Gln (see Table 1 for sequence). The last eluting peptide with *m/z* 921.5 was identified by CID (Figure 5B) and co-elution (Supplementary Figures 2I–K) as an octapeptide with the sequence pGlu-Leu-Thr-Phe-Ser-Thr-Gly-Trp amide and is named Dircl-AKH-II. It is identical to the first eight amino acids of the novel decapeptide found in *C. partellus*, Chipa-AKH (see below), which has the amino acid residues Gly and Asn in positions 9 and 10 to complete the decapeptide structure.

##### Papilionoidea: *Acraea horta*

The extracted base peak chromatograms of an extract from the CC of the garden Acraea *A. horta* exhibits four peaks representing AKHs at retention times of 5.18, 7.93, 8.08, and 8.16 min (Supplementary Figures 3A–D) corresponding, respectively, to (M + H)^+^ mass ions at *m/z* 1194.6, 1008.5, 907.4, and 1045.5, respectively (Supplementary Figures 3E–H). In some cases also the (M + NH_4_)^+^ ion can be seen, at *m/z* 1025.4 and 1062.4 (Supplementary Figures 3F,H, respectively), corroborating the identified mass. The first two eluting peaks, i.e., *m/z* 1194.5 and 1008.5, were subjected to CID and co-elution experiments to determine the peptide sequence; the data revealed the same peptide sequences as found in the CC sample of *D. clytus clytus* (see above), viz. the incompletely processed AKH (*m/z* 1194.6, Vanca-AKH) and Manse-AKH (*m/z* 1008.5; Supplementary Figure 3K top panel). For the third eluting peptide from the CC of *A. horta*, the CID spectrum of *m/z* 907.5 assigned this peptide clearly as an octapeptide with the sequence pGlu-Leu/Ile-Thr-Phe-Ser-Ser-Gly-Trp amide (Supplementary Figure 3I), which, with a Leu^2^ isomer, had previously been identified as Piebr-AKH (see section “Introduction” and Table 1). Co-elution with the synthetic peptide Piebr-AKH confirmed that *A. horta* contains this peptide (Supplementary Figure 3K middle panel). The last eluting AKH peptide of this extract at *m/z* 1045.5 was also easily identified from its CID spectrum (Supplementary Figure 3J). It is a nonapeptide with the sequence pGlu-Leu/Ile-Thr-Phe-Thr-Pro-Asn-Trp-Gly amide. Such a peptide, with Leu^2^, had been shown to exist in the kissing bug *Triatoma infestans* and had, therefore, been named Triin-AKH (Marco et al., 2013). Co-elution of the *A. horta* extract with the synthetic Triin-AKH confirmed that this peptide also occurs in the garden Acraea (Supplementary Figure 3K bottom panel).

##### Pyraloidea: *Chilo partellus*

**FIGURE 6:**
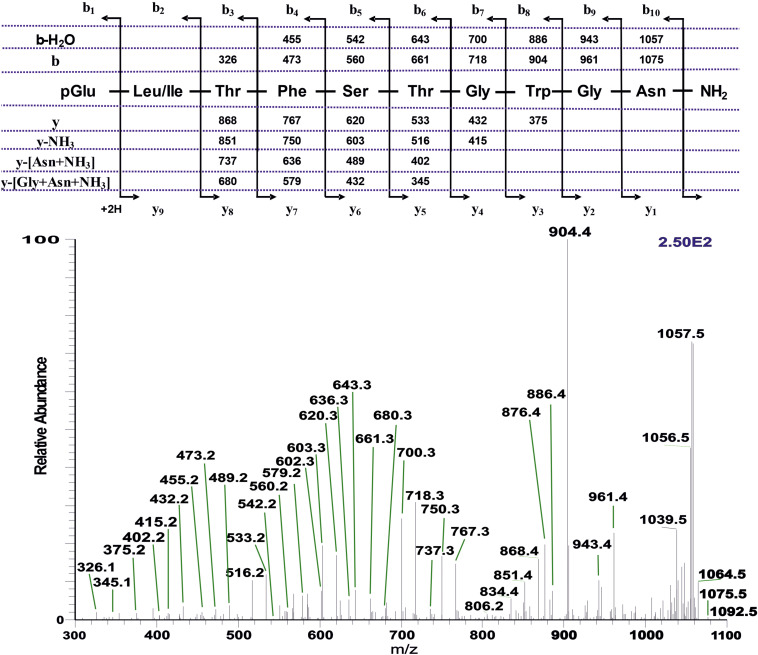
The CID tandem MS + ESI spectrum of the ion (M + H)^+^ at *m*/*z* 1092.5 (in Supplementary Figure 4D) from *Chilo partellus*. The inset shows the proposed peptide sequence together with diagnostic fragment ions observed in the MS^2^ spectra.

The base peak chromatogram (Supplementary Figure 4A) of an extract from the CC of the spotted stem borer *C. partellus* shows two AKH peaks. The extracted mass peaks of those are at 7.62 and 8.01 min (Supplementary Figures 4B,C) with the corresponding (M + H)^+^ mass ions at *m/z* 1092.5 (Supplementary Figure 4D, weak and embedded in other ions, see arrow) and *m/z* 1008.4 (Supplementary Figure 4E), respectively. The CID spectra gave clear product ions in both cases and led to the interpretation of a decapeptide AKH with the sequence pGlu-Leu/Ile-Thr-Phe-Ser-Thr-Gly-Trp-Gly-Asn amide (Figure 6) and a nonapeptide AKH with the sequence pGlu-Leu/Ile-Thr-Phe-Thr-Ser-Ser-Trp-Gly amide; the latter was shown to be the well-known Manse-AKH that occurs in the majority of Lepidoptera (see for example, Supplementary Figure 2B from *D. clytus clytus* CC; Supplementary Figure 3K from *A. horta* CC). By co-elution the Leu^2^ isomer was established for both peptides (Supplementary Figures 4F–H). The decapeptide (*m/z* 1092.5) is novel and is given the name Chipa-AKH; it has the same sequence as the novel Dircl-AKH-II but is extended by Gly at position 9 and Asn at position 10. Chipa-AKH is structurally closely related to Helze-HrTH with a conservative Ser to Thr substitution at position 6 (see Table 1).

##### Bombycoidea: *Actias luna*

**FIGURE 7:**
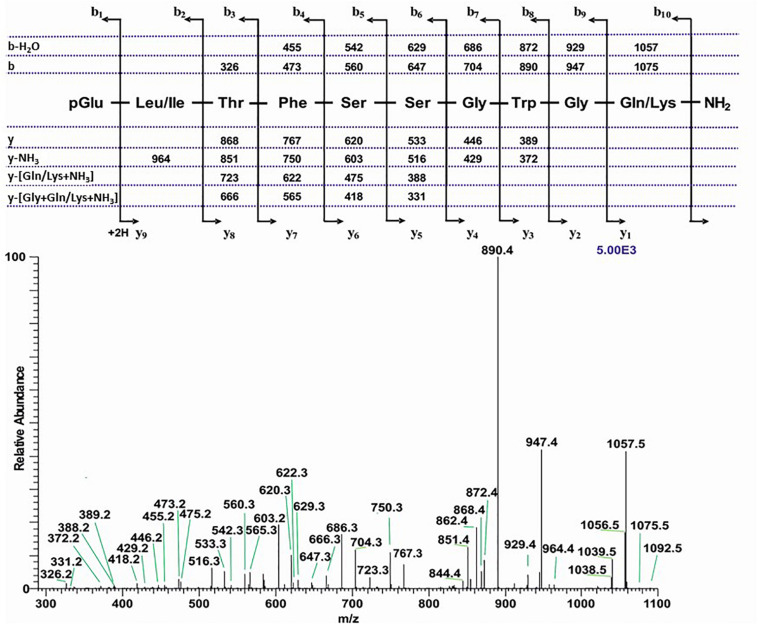
The CID tandem MS + ESI spectrum of the ion (M + H)^+^ at *m*/*z* 1092.5 (in Supplementary Figure 5C) from *Actias luna*. The inset shows the proposed peptide sequence together with diagnostic fragment ions observed in the MS^2^ spectra.

An extract from the CC of the luna moth *A. luna* of the family Saturniidae shows two ion peaks with retention time of 7.35 min and *m/z* 1092.5, and retention time of 8.03 min at *m/z* 1008.5 (Supplementary Figures 5A–D). In both cases further informative ions such as (M + NH_4_)^+^, (M + Na)^+^, and (M + NH_4_ + Na)^+^ were detected as well. Although the masses are the same as what was found in the CCs of *C. partellus* (see above), CID and co-elution experiments clearly show that there is a different peptide behind the (M + H)^+^ 1092.5 in *A. luna*: a sequence was assigned with two ambiguities due to isobaric amino acids at position 2 (Leu/Ile) and 10 (Gln/Lys; Figure 7). A peptide with the sequence pGlu-Leu-Thr-Phe-Ser-Ser-Gly-Trp-Gly-Gln amide, code-named Manse-AKH-II, is known to occur in several hawk moths (family: Sphingidae, see section “Introduction”); synthetic Manse-AKH-II was, therefore, used in a co-elution experiment and this clarified Leu at position 2 and Gln at position 10 of the *A. luna* peptide (Supplementary Figure 5E). Moreover, the elemental composition of a Gln residue is C_5_H_8_N_2_O_2_ = 128.0586 Da, while a Lys residue shows C_6_H_12_N_2_O = 128.0950 Da, thus, a difference of −0.0364 which is measurable by HRMS with Orbitrap mass spectrometry. Manse-AKH-II as an AKH in *A. luna* CC was corroborated when an exact mass of 1092.5109 was measured by HRMS with 0.8 ppm accuracy (*n* = 2); this corresponds to the elemental composition of C_50_H_70_N_13_O_15_ with Leu^2^ and Gln^10^ assigned to the sequence in Figure 7. An alternative peptide with Leu^2^ and Lys^10^ would have yielded MH^+^ 1092.5473 that fits with the elemental composition of C_51_H_74_N_13_O_14_.

Collision-induced dissociation and coelution (Supplementary Figure 5E) of the (M + H)^+^ ion at *m/z* 1008.5 led to the interpretation of pGlu-Leu-Thr-Phe-Thr-Ser-Ser-Trp-Gly amide as amino acid sequence, thus, Manse AKH is synthesized in *A. luna*.

##### Bombycoidea: *Antheraea yamamai*

**FIGURE 8:**
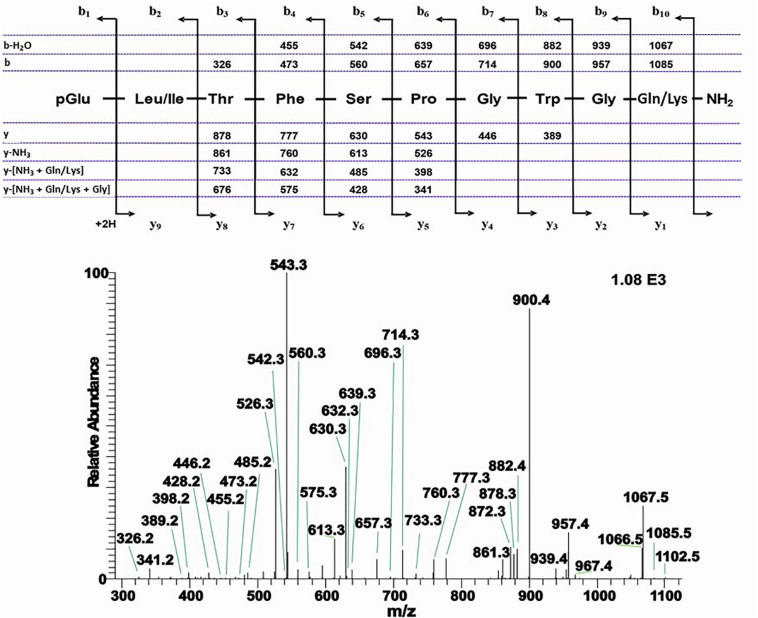
The CID tandem MS + ESI spectrum of the ion (M + H)^+^ at *m*/*z* 1102.5 (in Supplementary Figure 6B) from *A. yamamai*. The inset shows the proposed peptide sequence together with diagnostic fragment ions observed in the MS^2^ spectra.

An extract from the CC of the Japanese oak silk moth *A. yamamai* of the family Saturniidae shows two peaks with retention times of 7.64 and 7.95 min, respectively, which correspond with *m/z* 1102.5 and 1008.4, respectively (Supplementary Figures 6A–C). CID and co-elution experiments confirmed the well-known Manse-AKH for the latter (Supplementary Figures 6D–F) but the former led to the assignment of a novel decapeptide AKH for Lepidoptera. The CID assignment pointed to a peptide with two isobaric amino acids, a Leu or Ile residue at position 2 and a Gln or Lys residue at position 10 (Figure 8). A combination of co-elution with the synthetic compound having Leu^2^ and Gln^10^ (Supplementary Figures 6G–I), as well as LC-HRMS measurement (MH^+^ 1102.5316 in theory and 1102.5316 measured) led to the elemental composition of C_52_H_72_N_13_O_14_ and the sequence pGlu-Leu-Thr-Phe-Ser-Pro-Gly-Trp-Gly-Gln amide which we name Antya-AKH (Figure 8). This novel peptide is structurally most related to the decapeptide Bommo-AKH found in *B. mori*: a conservative Ser to Thr exchange has taken place at position 5.

#### AKHs of Lepidoptera Are (Almost) Exclusively Order-Specific

Extracts of CC from all other investigated Lepidoptera were treated the same as reported above, and the results of the assignment of their AKHs are collated in Table 1. By using ESI–MS methodology and comparison of various parameters of natural and synthetic peptide material, we have unequivocally identified the sequences of AKHs from 34 lepidopteran species of which the vast majority had not been investigated earlier. Four of these AKHs are novel and a further two have been found the first time in Lepidoptera but are known from other orders. Furthermore, we scanned databases for genomic/transcriptomic information on AKHs in Lepidoptera and have added those (predicted) AKH sequences, together with known lepidopteran AKHs from previously published reports (see section “Introduction”) into Table 1 to cover 76 lepidopteran species in total. Note that our efforts to scan genomes/transcriptomes did not always result in success, and such datasets, therefore, need to be taken with caution as it may be incomplete. Moreover, the interpretation of sequence processing from a gene/precursor does not take additional post-translational modifications of the peptide into account, other than cleavage from the precursor and amidation at the C-terminus. Cyclizing of the N-terminal Glu/Gln is inferred from sequence homology with biochemically sequenced AKHs, but there are several other post-translation modifications that are known to occur in AKHs – as gleaned from sequencing the mature peptides (e.g., hydroxprolination, C-mannosylation of Trp, modification of Trp to kynurenine, sulfation, phosphorylation; see Marco and Gäde, 2020). Hence, predicted AKH sequences are just that: predictions and the sequence of the mature peptide remains to be confirmed by biochemical means; as such, we have given code names only to confirmed mature AKH sequences in the current study. Despite the shortcomings of only using bioinformatic searches, we have identified two more decapeptides that seem to be novel: in *Eumeta japonica* (superfamily: Tineoidea; pELTFTSNWGS amide) and in *Ostrinia furnacalis* (superfamily: Pyralidae; pELTFSTGWGQ amide). Once these putative AKHs have been shown, through mass spectrometry, to occur as mature peptides in the CC, they will be given their respective code names. Further, to orientate the reader, the data in Table 1 are presented in a phylogenetic order (see Mitter et al., 2017; Kawahara et al., 2019; Figure 1).

When assessing this impressively large data set of 76 studied species in Table 1, we can reach a number of conclusions:

1. The maximal number of AKHs (five) expressed in the CC of the sphingid moth genus *Hippotion* (Gäde et al., 2013) is very likely a single evolutionary event. No other species investigated here has more than 2 or 3 AKHs (Table 1). Three AKHs are found in quite a few species of the family Noctuidae. Most other families have two AKHs. Interestingly, the most basal families of which we have data so far (from the superfamilies Adeloidea and Tineoidea) appear to have only one AKH, although it must be cautioned that this information was mined from genomic data which may have been incomplete or not completely accessible during our searches.
2. In total there are 15 different AKHs in Lepidoptera known to date from biochemical characterization – not counting the incompletely processed, inactive form of Manse-AKH (i.e., Vanca-AKH): six decapeptides, four nonapeptides and five octapeptides; a further two novel decapeptide sequences are predicted. This size distribution is quite different to what is seen in another speciose order, the Diptera, where 14 different AKHs are known to date – all are octapeptides, bar one (Gäde et al., 2020). Most interestingly, nonapeptides are a feature of Lepidoptera: the only other nonapeptide outside the Lepidoptera is found in the Hemiptera (a kissing bug; Marco et al., 2013); this same nonapeptide is identified in the current study in a nymphalid butterfly, *A. horta*.
3. Almost all lepidopteran species studied contain the nonapeptide Manse-AKH. This is certainly a signature peptide for the order Lepidoptera. It seems, however, to be absent in the more basal superfamilies represented in this study by Adeloidea, Tineoidea, Yponomeutoidea, and Tortricoidea and “emerges” with the Papilionoidea and is thereafter present in all phylogenetically “younger” superfamilies.
4. The basal superfamilies (mentioned above), on the other hand, all contain the octapeptide Peram-CAH-II, or the novel peptide predicted for *E. japonica* which differs from Peram-CAH-II at position 6 by a Ser instead of a Pro residue and in the chain length (octa- versus decapeptide). Peram-CAH-II is a peptide well-known from blattid cockroaches (order: Blattodea), from certain Caelifera (order: Orthoptera), Heteroptera (order: Hemiptera), and Chrysomeloidea (order: Coleoptera) (Gäde, 2009; Gäde et al., 2019) and recently, amplified and sequenced after data mining the codling moth transcriptome (Garczynski et al., 2019). From the superfamily Yponomeutoidea onward, including the superfamily Tortricoidea, we find also the decapeptide Lacol-AKH which then re-emerges in the superfamily Noctuoidea.
5. The octapeptide Piebr-AKH seems to be characteristic for the superfamily Papilionoidea (and also found in Gelechioidea) but has a scattered distribution in the various families: only one member of the Pieridae families and most species of the Nymphalidae produce Piebr-AKH together with Manse-AKH), whereas Papilionidae, Hesperiidae, and Lycaenidae do not. The latter families predominantly have Manse-AKH as sole AKH peptide, of those, four of the species were investigated by mass spectrometry and only Manse-AKH was detected and confirmed, while for the majority of species from these families, a bioinformatics search could only reveal Manse-AKH. Only two species of investigated Nymphalidae do not produce the common pair of AKHs (Manse-AKH + Piebr-AKH), or only Manse-AKH, viz. *D. clytus clytus* and *A. horta*. *D. clytus clytus* has a second nonapeptide – the novel Dircl-AKH-I – in addition to Manse-AKH, plus a novel octapeptide, Dircl-AKH-II, which differs from Piebr-AKH by a Ser/Thr exchange at position 5. *A. horta*, has, in addition to Manse-AKH and Piebr-AKH, also recruited a nonapeptide (Triin-AKH).
6. The one investigated species in the superfamily Gelechioidea, *Atrijuglans hetaohei*, produces Manse-AKH and Piebr-AKH (see Li et al., 2020). Apparently, there is still much controversy and discussion about the taxonomic status and the phylogenetic position of *A. hetaohei*: Chinese scholars have widely applied the proposal that *A. hetaohei* is a member of Heliodinidae (superfamily Yponomeutoidea) (see Wang et al., 2016). This is, however, not the same conclusion drawn based on morphology and comparisons of mitochondrial genomes of Lepidoptera (Wang et al., 2016). Based on the predicted AKHs of *A. hetaohei* as deduced from transcriptome screening from heads of the moth (Li et al., 2020), we agree that *A. hetaohei* should be placed into family Stathmopodidae (superfamily Gelechioidea). Manse-AKH and Piebr-AKH both first appear in the superfamily Papillionoidea and cannot be reconciled to such an early appearance as in the superfamily Yponomeutoidea.
7. The superfamily Pyraloidea has as characteristic AKH the octapeptide Chipa-AKH (mostly in addition to the signature peptide Manse-AKH) or the novel peptide found in *O. furnacalis* which differs from Chipa-AKH only in residue 10 (a conservative Asn/Gln exchange).
8. The superfamily Noctuoidea contains always in addition to Manse-AKH the decapeptide Helze-HrTH, as shown previously (Jaffe et al., 1988). Thus, Helze-HrTH is certainly characteristic for this superfamily. Occasionally a third AKH can be found, Lacol-AKH (Gäde et al., 2008). Lacol-AKH, together with Helze-HrTH is also present in *Mythimna separata* (current study) and *Agrotis ipsilon* (Diesner et al., 2018). In studying the datasets of Diesner et al. (2018), we realize that the authors overlooked Manse-AKH in *A. ipsilon*: in their interpretation of putative preprohormones from transcriptomic information, the authors grouped the Manse-AKH precursor together with the Lacol-AKH precursor as “adipokinetic 1” (see data in Table 2 of Diesner et al., 2018). Evidence for the presence of Manse-AKH in CC tissue of *A. ipsilon* is shown (but not acknowledged/interpreted) in Figure 4B of Diesner et al. (2018) where the sodium adduct of Manse-AKH is clearly visible as mass ion 1030.47, along with the sodium adducts of Lacol-AKH (1087.49) and Helze-HrTH (1100.48). With our reinterpretation, thus, we have added Manse-AKH to the complement of AKHs in this noctuid moth species.
9. In the superfamily Bombycoidea, the three investigated families Saturniidae, Bombycidae, and Sphingidae contain besides the nonapeptide Manse-AKH, a decapeptide with the C-terminal tetrapeptide -GWGQ amide. Whereas Manse-AKH-II is found in all Sphingidae (except *Smerinthus ocellata*) and in one species of the Saturniidae, the other saturniid moth has the closely related Antya-AKH (Ser^6^/Pro^6^ exchange) and the bombycid moth has Bommo-AKH which differs from Antya-AKH by a Ser^5^/Thr^5^ substitution.
10. As a point of interest, one has to mention that no Val or Ile (normally at position 2) is found in any member of the AKHs of Lepidoptera, unlike in Diptera (Gäde et al., 2020).

Thus, we can conclude that the data as presented to date in Table 1 point largely to an order-specificity and to some specificity in the superfamilies. In Nymphalidae where we have a relatively large data set with 19 studied species, no further differentiation between subfamilies can be made.

### Adipokinetic Response of Lepidoptera AKH Family Bioanalogs: Potential for “Green” Insecticides?

We have already mentioned the dichotomous status of Lepidoptera as harmful (pest) and beneficial insects. Caterpillars of many lepidopteran species are assumed major agricultural pest species where they may be feeding mainly on flowers and reproductive structures of a single plant family or are polyphagous as a number of heliothine moths (Cunningham and Zalucki, 2014). The caterpillars may defoliate trees or whole forests or they can be devastating in destroying stored foods (see, Xu et al., 2016). Certain adult Lepidoptera (notably moths) are damaging too, by eating textiles (Basuk and Behera, 2018). Hence, it would be desirable to manage and fight such lepidopteran species/life stages specifically. Targeting insect neuropeptides and their cognate receptors (GPCRs) have been proposed as a good strategy for this task in integrated pest management (Audsley and Down, 2015; Verlinden et al., 2014). We assess here the potential of the AKH family of peptides. Whereas in a first step the complement of existing AKH peptides has to be determined, subsequent studies will aim to build a model of ligand-receptor binding, as has already been proposed for the AKH systems of the Malaria mosquito *Anopheles gambiae* (Mugumbate et al., 2013), the desert locust *Schistocerca gregaria* (Jackson et al., 2019) and the blowfly *Phormia terraenovae* (Abdulganiyyu et al., 2020b), as well as the red pigment-concentrating hormone system of the water flea, *Daphnia pulex* (Jackson et al., 2018). Thereafter, peptide mimetics will be tested (Altstein and Nässel, 2010) in a cellular receptor assay system (see, for example, in Caers et al., 2012; Abdulganiyyu et al., 2020a).

In the current study we have compiled a list of all the known Lepidoptera AKHs to date and went one step further to investigate ligand-receptor interaction of these AKH bioanalogs via a well-established *in vivo* bioassay in which the adipokinetic effect of an injected substance is measured in the hemolymph of a resting insect. The cabbage butterfly *P. brassicae* is the acceptor insect and all 15 biochemically confirmed, fully processed Lepidoptera AKH structures were tested (see Table 3). Previously, the incompletely processed AKH (Vanca-AKH) was tested in *P. brassicae* and found to have no adipokinetic activity (Marco and Gäde, 2017). The 15 chemically synthesized peptides were quantified via RP-HPLC and diluted in distilled water for injection, hence, distilled water served as a negative control substance to see that the solvent and handling of the animal did not artifactually increase the lipid concentration in the hemolymph. Both male and female test insects were used less than 48 h post-emergence and a mean lipid concentration of 22.22 ± 6.24 μg/μl (*n* = 123) was measured at the start of the experiment (Supplementary Table 1). A dose of 10 pmol peptide was injected as this previously gave unambiguous hyperlipemic results in *P. brassicae* (Marco and Gäde, 2017). The bioassay results are unequivocal (Table 3 and Supplementary Table 1): distilled water and the handling/injection of butterflies do not elicit a stress (hyperlipemic) effect in the test insect, in fact, a slight dip in lipids (not significant) is recorded; two AKH bioanalogs fail to increase circulating lipids significantly in *P. brassicae* viz. Peram-CAH-II and Bommo-AKH, while Antya-AKH has a very small adipokinetic effect. One of the other low-performers is Triin-AKH; what these four peptides have in common is a Pro residue in position 6. One could speculate that Triin-AKH performed best in this group because it is a nonapeptide, which is a hallmark of Lepidoptera AKHs. Interestingly, when a Lacol-AKH analog was synthesized with Pro^6^ and injected into *H. eson* adults in an earlier study (Marco and Gäde, 2015), the adipokinetic response was only slightly reduced to 94% of the possible maximal response. It may be too early to draw sound conclusions based only on experimentation with two species, but it seems as if the Pro^6^ may differentially affect receptor-ligand interaction in AKH signaling in Lepidoptera. It can be predicted that *H. eson* would respond positively to the Pro-containing AKHs that performed poorly (or not at all) in *P. brassicae*. One could then speculate that a peptide mimetic based on a lead AKH peptide with Pro^6^, may lend some specificity in its action amongst the Lepidoptera.

**TABLE 3:** The difference in circulating lipid concentration following injection of distilled water or a synthetic Lepidoptera AKH peptide (10 pmol) into the cabbage white butterfly, *Pieris brassicae*.

**Injectate/amino acid sequence**	***n***	**Difference (μg/μl)**	***P***
Distilled water	10	−0.44 ± 2.87	NS
Peram-CAH-II: pELTFTPNWa	9	1.12 ± 2.57	NS
Bommo-AKH: pELTFTPGWGQa	6	0.94 ± 2.39	NS
Antya-AKH: pELTFSPGWGQa	8	1.80 ± 1.48	0.005
Dircl-AKH-II: pELTFSTGWa	10	3.60 ± 2.44	0.0006
Triin-AKH: pELTFTPNWGa	6	3.69 ± 1.71	0.002
Hipes-AKH-III: pELTFTSTWGa	9	3.77 ± 1.63	0.00006
Hipes-AKH-I: pELTFTSSWa	9	3.80 ± 1.58	0.00005
Manse-AKH-II: pELTFSSGWGQa	8	4.24 ± 1.18	0.00001
Helze-HrTH: pELTFSSGWGNa	7	4.56 ± 1.16	0.00002
Piebr-AKH: pELTFSSGWa	7	4.69 ± 2.05	0.0005
Manse-AKH: pELTFTSSWGa	6	4.70 ± 2.51	0.003
Dircl-AKH-I: pELTFSSGWGa	6	5.13 ± 1.77	0.0004
Lacol-AKH: pELTFTSSWGGa	7	5.18 ± 2.25	0.0004
Hipes-AKH-II: pELTFTSTWa	8	6.47 ± 1.63	0.00001
Chipa-AKH: pELTFSTGWGNa	7	8.28 ± 2.98	0.0002

*Data arranged in increasing order of response.*

The remaining AKH bioanalogs tested currently in *P. brassicae* can be arbitrarily divided into different groups based on the resulting metabolic increase after injection. For example, in the lower-performing group are the octapeptides Dircl-AKH-II and Hipes-AKH-I, as well as the nonapeptide Hipes-AKH-III. None of these three peptides have a Pro residue; in the case of Hipes-AKH-I one could make two arguments (i) that peptide length is the decider on receptor interaction when the structure of Hipes-AKH-I is compared with that of one of the endogenous *P. brassicae* peptides (Manse-AKH): these peptides are near-identical save for the Gly^9^ residue in Manse-AKH, and (ii) Hipes-AKH-I has two amino acid substitutions when compared with the endogenous octapeptide of *P. brassicae* Piebr-AKH, one of which is a Thr residue in position 5 instead of a Ser. The difference between Manse-AKH and Hipes-AKH-III also revolves around the seemingly simple, innocuous substitution of Ser and Thr in position 7, and the substitution of Ser and Thr is also involved between Piebr-AKH and Dircl-AKH-II but this time at position 6 (see Table 3). In all of these cases, the substituted Thr seems to be less-favored for endocrine signaling in the cabbage white butterfly, however the adipokinetic result with Chipa-AKH (a decapeptide with Thr^6^) and Hipes-AKH-II (an octapeptide with Thr^7^) are then enigmatic in that these peptides elicited the highest increase in lipid concentration after injection in the bioassay series. Chemical modeling of the AKH ligand-receptor interactions and NMR studies with the Lepidoptera ligands may shed light on the *in vivo* observations and explain the overall peptide conformation and how this affects interaction with the binding pocket of the different lepidopteran AKH receptor when amino acids are substituted in positions 5, 6, and/or 7.

The good adipokinetic response in *P. brassicae* achieved with the novel nonapeptide Dircl-AKH-I, and the decapeptides Lacol-AKH, Manse-AKH, Manse-AKH-II and Helze-HrTH are all consistent with the above observation that Ser^6^Ser^7^, and/or Ser^5^Ser^6^ seems to be preferred in the ligand for interaction with the *P. brassicae* AKH receptor. That such conservative exchanges of Thr/Ser at positions 5, 6, or 7 would influence the endocrine result is surprising because the chemical properties of the two amino acid residues are similar: both are polar amino acids, although Ser is more polar since Thr has an extra methyl group; Pro on the other hand is hydrophobic. *In vivo* bioassays with *H. eson* as test insect, however, also hinted that the AKH receptor has a slight preference for ligands with Ser^7^ (Hipes-AKH-I, Manse-AKH, Lacol-AKH) or Gly^7^ (Manse-AKH-II) over those with Thr^7^ (Hipes-AKH-II and -III) (Gäde et al., 2013).

Can the current study provide useful pointers on the potential of the “green” insecticide concept with Lepidoptera AKHs? We see that Manse-AKH is a unique peptide present in almost all species of Lepidoptera but in no other order. It can be used as a lead peptide, and the chances that a final mimetic is specific only for Lepidoptera would then be high. Receptor binding studies have to be performed to ascertain whether further specificity is possible by making use of the various, superfamily specific, second AKHs. At first glance, the structures of these second AKHs seem not to be sufficiently different from Manse-AKH yet at the *in vivo* level, already small differences/trends can be seen in activity (current study). Previous receptor assay results with dipteran receptors and their endogenous AKHs (which are very similar in structure), also revealed subtle activation differences, giving hope that specificity on a case by case basis can be tested (Caers et al., 2012). Since a number of genomic studies on Lepidoptera have been carried out (Triant et al., 2018), which facilitates the cloning of more Lepidoptera AKH receptors, it is hoped that the modeling of ligand-receptor interaction and testing receptor activation may be achieved for some key lepidopteran species. It also remains to be seen whether Manse-AKH can function in the primitive Lepidoptera species where Peram-CAH-II is the endogenous AKH peptide.

### Putative Molecular Evolution of Lepidopteran AKHs

**FIGURE 9:**
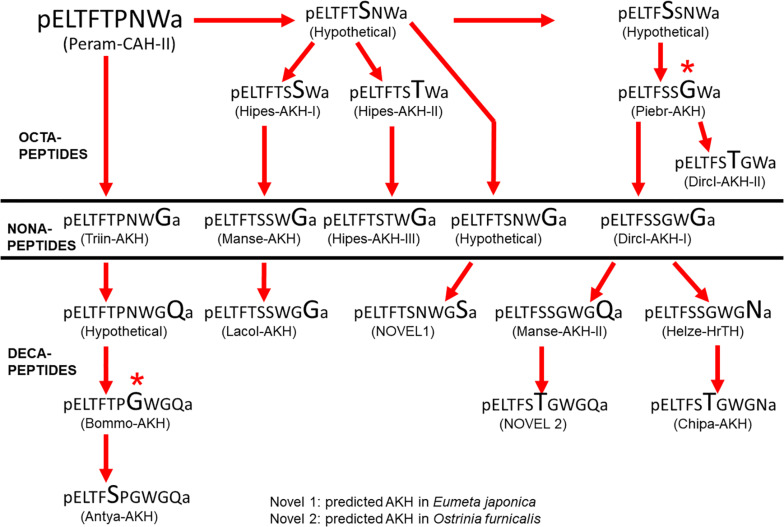
Hypothetical molecular evolution of adipokinetic peptides in Lepidoptera. Peram-CAH-II is assumed here as ancestral peptide for this order. The amino acid substitution in each peptide is indicated in a larger font than in the peptide from which it is hypothetically derived. All substitutions are point mutations (except * for the proposed change from Asn to Gly) and four hypothetical peptide sequences are proposed to enable such a molecular evolution.

A pre-requisite for the speculation of the molecular evolution of AKHs in Lepidoptera is the knowledge about a possible ancestral AKH of this order. This could probably be achieved by considering the structure of the AKH of the closest relatives to the Lepidoptera. Without doubt the caddisflies (Trichoptera) are assumed to be the sister group to Lepidoptera (Kristensen et al., 2007; Mitter et al., 2017). Unfortunately, at present there is no published AKH structure from any member of the Trichoptera. Thus, our next best choice is to look at the AKH(s) present in the most basal lepidopteran superfamily, the Micropterigoidea, but we have neither collected any species nor have we mined any AKH sequence from this superfamily. As outlined in Figure 1 we do, however, have data from a member of the family Adelidae (superfamily: Adeloidea) which has the octapeptide Peram-CAH-II predicted and a member of the family Psychidae (superfamily: Tineoidea) is predicted to have a very similar decapeptide (see Table 1). Since Peram-CAH-II has also been found in other orders and is the shorter of the two peptides, we assume here that this peptide is ancestral to Lepidoptera. As depicted in Figure 9 one can construct a tree for the possible molecular evolution of the Lepidoptera AKHs, taking into account that we need (i) a few hypothetical peptides which may exist in the vast numbers of not-yet sequenced lepidopteran species, (ii) an elongation from octa- to nona- and then decapeptides, and (iii) in two cases, two nucleotides of the triplet (instead of just a single base change) were expected to have mutated to accommodate the change from an Asn to a Gly residue. Future work will confirm, refute or modify this hypothetical scheme.

## Data Availability Statement

The datasets presented in this study can be found in online repositories. The names of the repository/repositories and accession number(s) can be found in the article/Supplementary Material.

## Author Contributions

GG: concept and design of the study, acquisition of insect species and synthetic peptides, breeding of certain species from eggs, data acquisition (biological assays), interpretation and analysis of data, and writing the draft manuscript. HM: co-designed the study, data acquisition (biological assays, dissection of insect corpora cardiaca and preparation of extracts, and mining data bases for AKH sequences), interpretation and analyses of the data, and writing and refining the draft manuscript. PŠ: mass spectrometric analyses, data interpretation, and drafting of MS figures for the manuscript. All authors contributed to the article and approved the submitted version.

## Conflict of Interest

The authors declare that the research was conducted in the absence of any commercial or financial relationships that could be construed as a potential conflict of interest.

## Footnotes

1 https://www.nhm.ac.uk/discover/record-breaking-butterflies.html

2 http://lepbase.org/

3 https://blast.ncbi.nlm.nih.gov/

4 https://gd.eppo.int/download/doc/133_datasheet_HELIZE.pdf
