# Supplementary material for: Unique Members of the Adipokinetic Hormone Family in Butterflies and Moths (Insecta, Lepidoptera)
Source: Front Physiol. 2020 Dec 17;11:614552. doi: 10.3389/fphys.2020.614552 (PMC7773649; doi:10.3389/fphys.2020.614552)
Supplement: Supplementary file 1 [file Data_Sheet_1.PDF]

SUPPLEMENTARY TABLE 1: LIPID BIOASSAYS IN *Pieris brassicae*

Adult *P. brassicae* specimens were used on the day of emergence or 1 day after emergence. 0.5 µl haemolymph was collected before and 90 min after injection of a test solution in 3 µl volume. Haemolymph samples in 100 µl H_2SO_4 were processed with 1 ml vanillin and absorption measured at 546nm in a glass cuvette. Paired t-test was performed to check for significant change in metabolite following injection.

Injectate (3 µl)	n	[lipid] T_{0min} (µg/µl)	[lipid] T_{90min} (µg/µl)	Difference (µg/µl)	P
Distilled water	10	25.62 ± 5.42	25.18 ± 5.58	-0.44 ± 2.87	NS
10 pmol Piebr-AKH	7	25.07 ± 4.20	29.76 ± 3.72	4.69 ± 2.05	0.0005
10 pmol Manse-AKH	6	22.29 ± 3.06	26.99 ± 4.00	4.70 ± 2.51	0.003
10 pmol Triin-AKH	6	20.39 ± 4.75	24.08 ± 5.71	3.69 ± 1.71	0.002
10 pmol Manse-AKH-II	8	18.50 ± 3.52	22.74 ± 3.89	4.24 ± 1.18	0.00001
10 pmol Helze-HrTH	7	20.46 ± 4.64	25.37 ± 4.26	4.56 ± 1.16	0.00002
10 pmol Chipa-AKH	7	29.51 ± 6.35	37.79 ± 8.70	8.28 ± 2.98	0.0002
10 pmol Antya-AKH	8	26.48 ± 5.17	28.28 ± 5.33	1.80 ± 1.48	0.005
10 pmol Lacol-AKH	7	20.76 ± 4.95	25.95 ± 3.23	5.18 ± 2.25	0.0004
10 pmol Bommo-AKH	6	22.25 ± 5.38	23.19 ± 3.35	0.94 ± 2.39	NS
10 pmol Peram-CAH-II	9	22.67 ± 7.03	23.79 ± 6.16	1.12 ± 2.57	NS
10 pmol Dircl-AKH-I	6	27.24 ± 8.76	32.37 ± 7.68	5.13 ± 1.77	0.0004
10 pmol Dircl-AKH-II	10	26.81 ± 3.96	30.41 ± 5.24	3.60 ± 2.44	0.0006
10 pmol Hipes-AKH-I	9	18.76 ± 4.97	22.56 ± 4.15	3.80 ± 1.58	0.00005
10 pmol Hipes-AKH-II	8	14.21 ± 2.79	20.68 ± 3.52	6.47 ± 1.63	0.00001
10 pmol Hipes-AKH-III	9	16.58 ± 2.49	20.35 ± 2.45	3.77 ± 1.63	0.00006
