# Supplementary material for: Unique Members of the Adipokinetic Hormone Family in Butterflies and Moths (Insecta, Lepidoptera)
Source: Front Physiol. 2020 Dec 17;11:614552. doi: 10.3389/fphys.2020.614552 (PMC7773649; doi:10.3389/fphys.2020.614552)
Supplement: Supplementary file 2 [file Data_Sheet_2.PDF]

Lepidoptera AKHs

SUPPLEMENTARY FIGURE S1: Confirmation of the AKH peptide structures in the codling moth *C. pomonella* corpus cardiacum extract by HPLC-MS co-elution of the two native peaks with the corresponding diluted synthetic AKH peptide.

FIG. S1A-C. An LC-MS co-elution experiment of the CC extract from the codling moth spiked with the synthetic peptides pELTFTSSWGG amide (Lacol-AKH; MH^+ = 1065.5) and pELTFTPNW amide (Peram-CAH-II; MH^+ = 988.5). **S1A** and **B** show the individual diluted synthetic peptides Lacol-AKH and Peram-CAH-II, respectively, whereas **S1C** depicts the extract spiked with the two synthetic peptides. In each case only one prominent peak is revealed indicating that the amino acid in position 2 is Leu and not Ile.

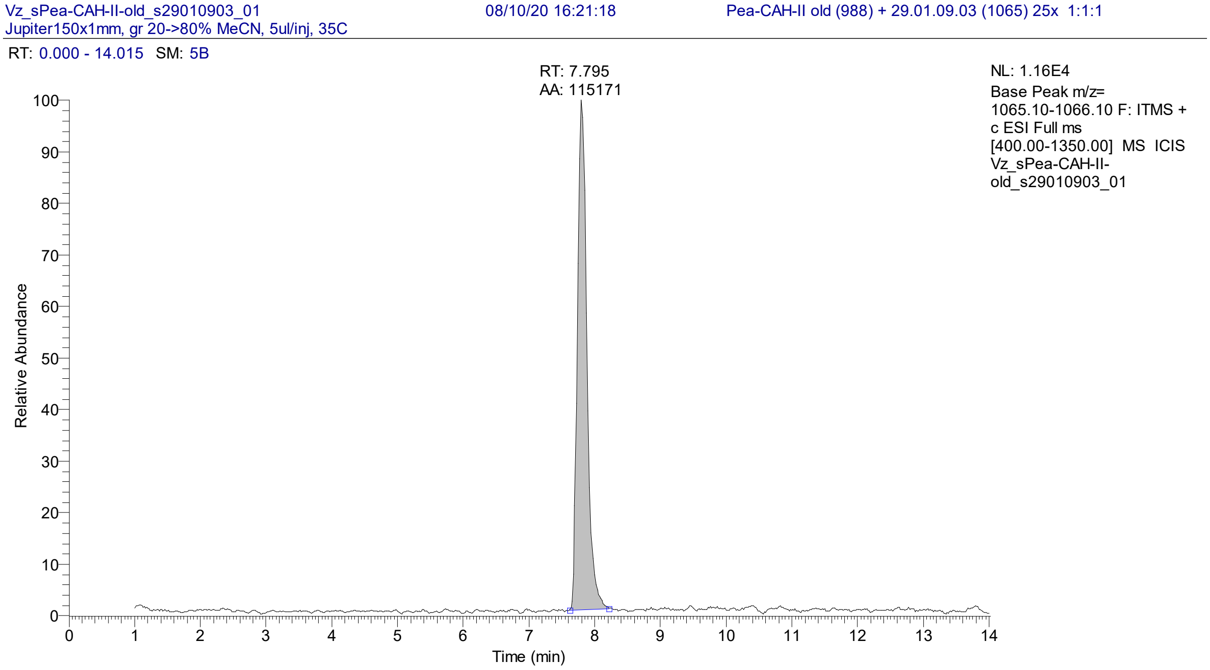

Fig. S1A. A base peak LC-MS chromatogram of the synthetic Lacol-AKH (MH^+ = 1065.5)

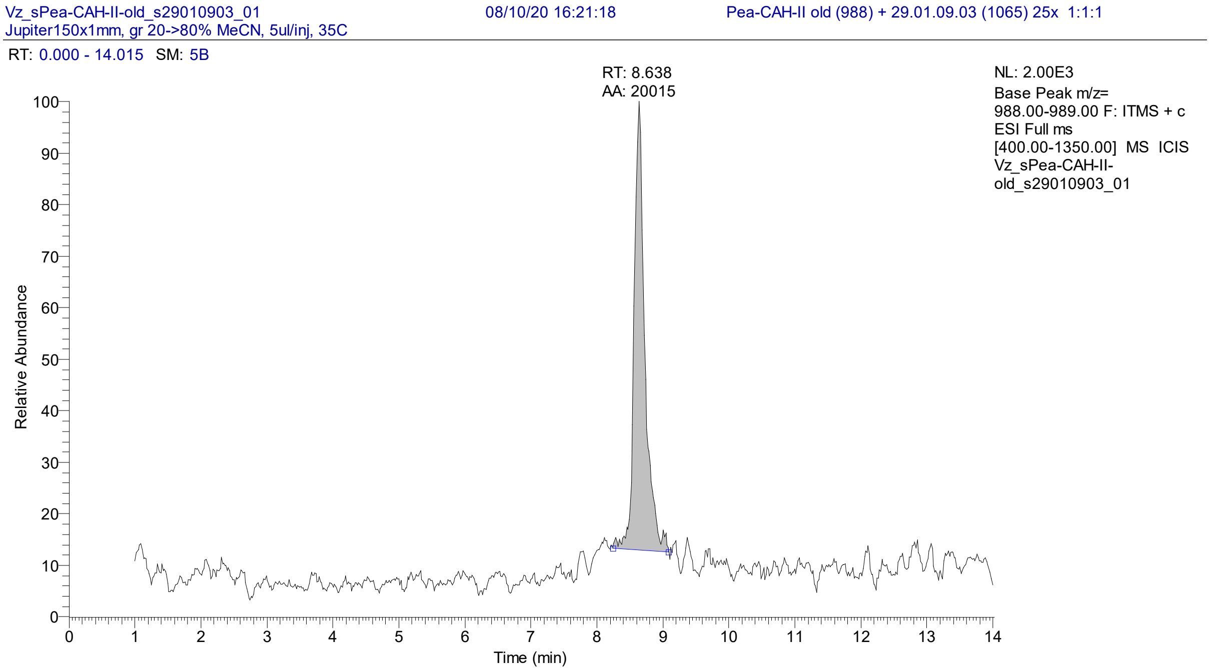

Fig. S1B. A base peak LC-MS chromatogram of the synthetic Peram-CAH-II (MH$^+$ = 988.5).

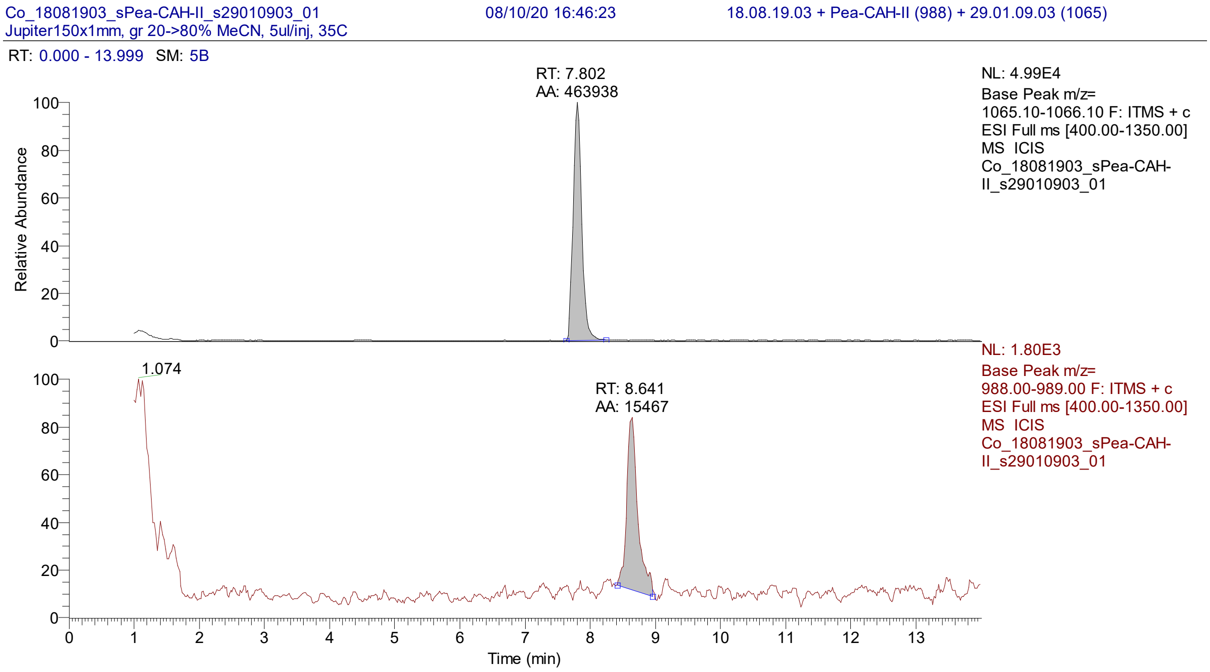

Fig. S1C. LC-MS extracted chromatogram of the codling moth CC extract spiked with synthetic Lacol-AKH (top) and Peram-CAH-II (bottom). Native and synthetic peaks elute together and are proof for the correct sequence assignment.

SUPPLEMENTARY FIGURE S2: Collision-induced dissociation (CID) tandem MS+ESI spectra. **(A)** The CID spectrum of the ion $[M + H]^+$ at *m*/*z* 1194.6 in **FIGURE 4B** from *Dira clytus clytus*. **(B)** The CID spectrum of the ion $[M + H]^+$ at *m*/*z* 1008.5 in **FIGURE 4D** from *D. clytus clytus*. The insets show the proposed peptide sequences together with diagnostic fragment ions observed in the MS^2 spectra.

Fig. S2 C-E. Confirmation of the AKH peptide structures in the Cape autumn widow *D. clytus* corpus cardiacum extract by HPLC-MS co-elution of the three native peaks with the corresponding diluted synthetic AKH peptide.

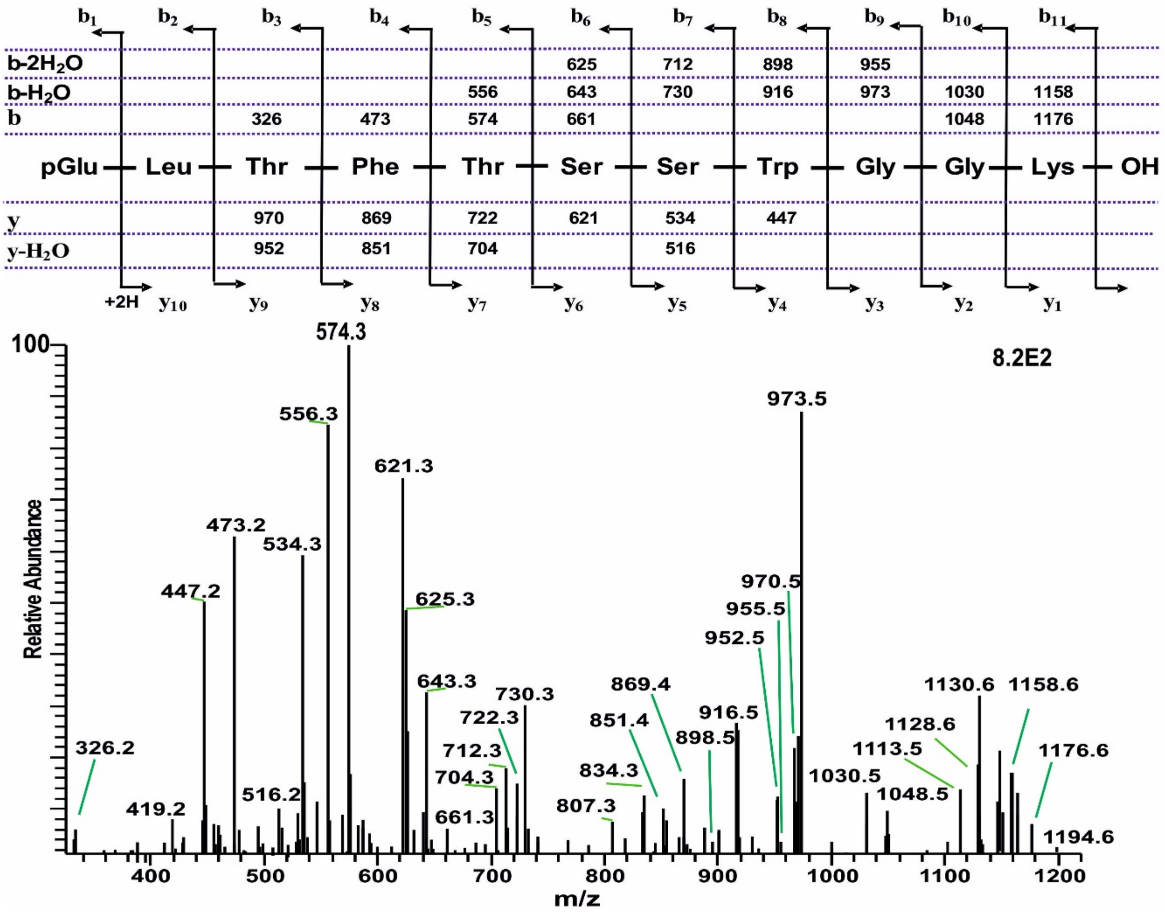

Fig. S2A. A CID tandem MS+ESI spectrum of the ion $[M + H]^+ = 1194.6$ in Figure 4B from the CC of *D. clytus.*

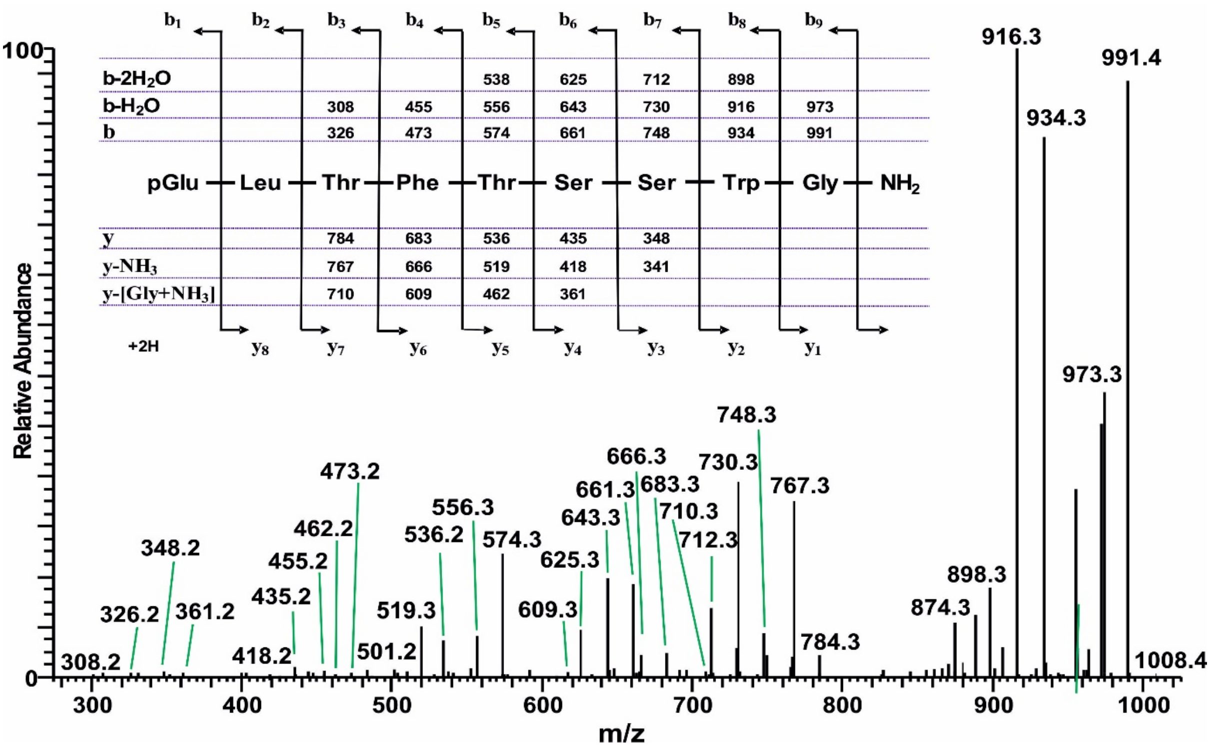

Fig. S2 B. A CID tandem MS+ESI spectrum of the ion $[M + H]^+ = 1008.4$ in Figure 4D from the CC of *D. clytus*.

Fig. S2 C-E. An LC-MS co-elution experiment of the CC extract-derived peptide with MH$^+$ 1008.4 from the *D. clytus clytus* CC spiked with the synthetic peptide: pELTFTSSWG amide (Manse-AKH; MH$^+$ = 1008.5). The chromatograms C-E reveal one prominent peak that co-incides with the retention time of the native peptide, thus indicating that the amino acid in position 2 is Leu, and not Ile.

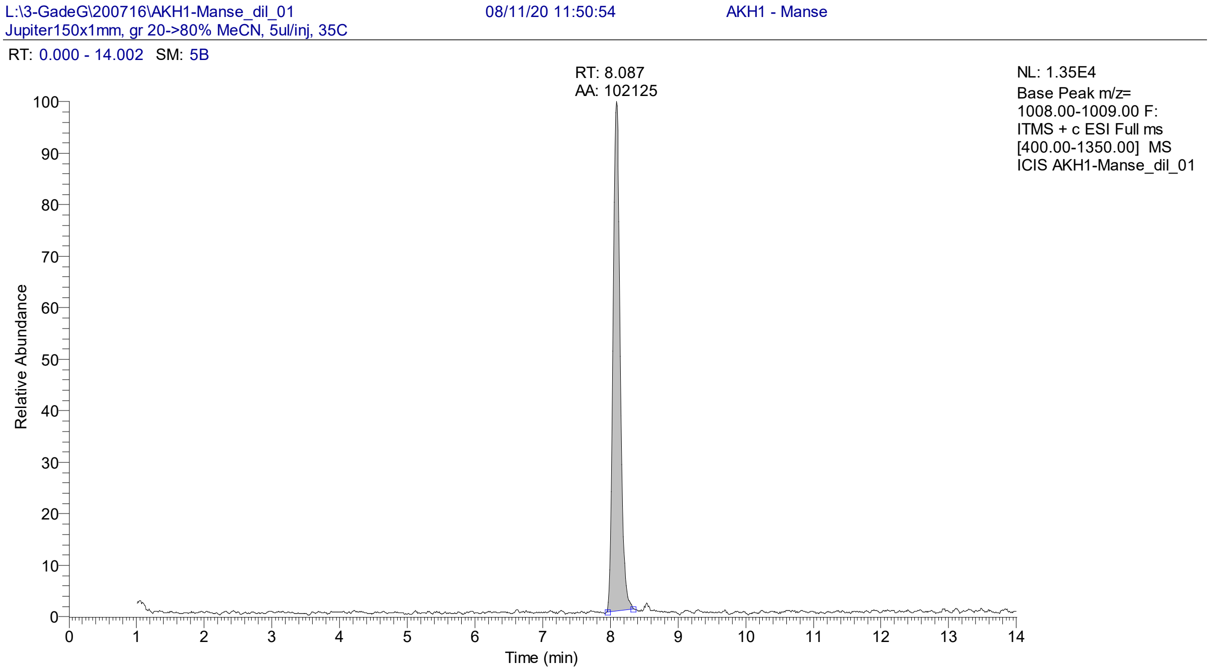

Fig. S2C. A base peak LC-MS chromatogram of the synthetic AKH peptide Manse-AKH pELTFTSSWG amide (MH$^+$ = 1008.4).

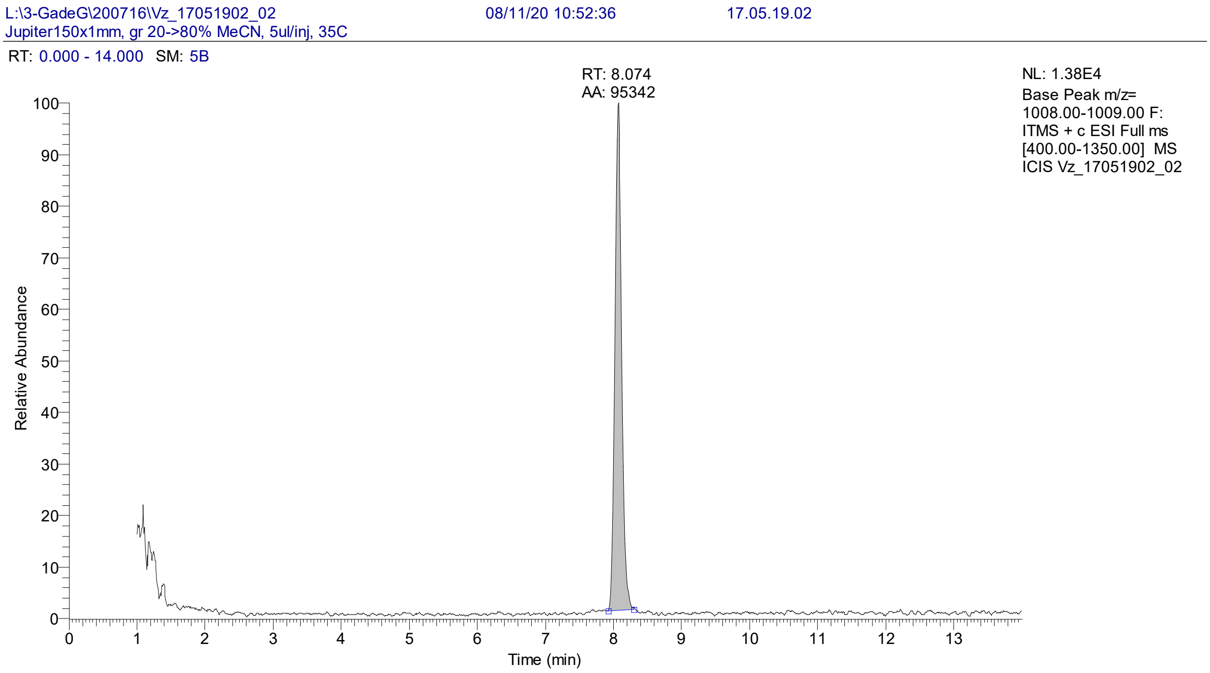

Fig. S2D. A base peak LC-MS chromatogram of the detected AKH with MH$^+$ = 1008.4 from the *D. clytus clytus* CC.

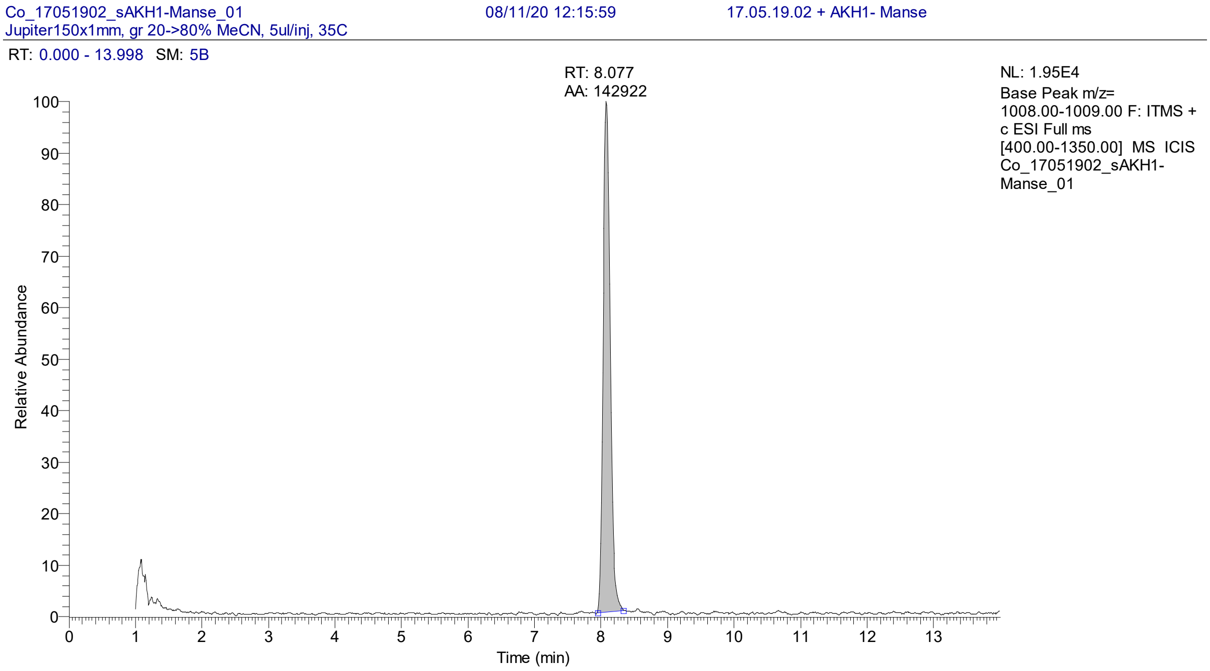

Fig S2 E. A base peak LC-MS chromatogram of the detected AKH with MH^+ = 1008.4 from the *D. clytus clytus* CC spiked with the synthetic Manse-AKH peptide. The native peak co-elutes with the synthetic peptide and thus proves Leu at position 2.

Fig. S2 F-H. An LC-MS co-elution experiment of the CC extract-derived peptide with MH^+ 964.4 from the *D. clytus clytus* CC spiked with the synthetic peptide: pELTFSSGWG amide (Dircl-AKH-I; MH^+ = 964.4). The chromatograms F-H reveal one prominent peak that co-incides with the retention time of the native peptide, thus indicating that the amino acid in position 2 is Leu, and not Ile.

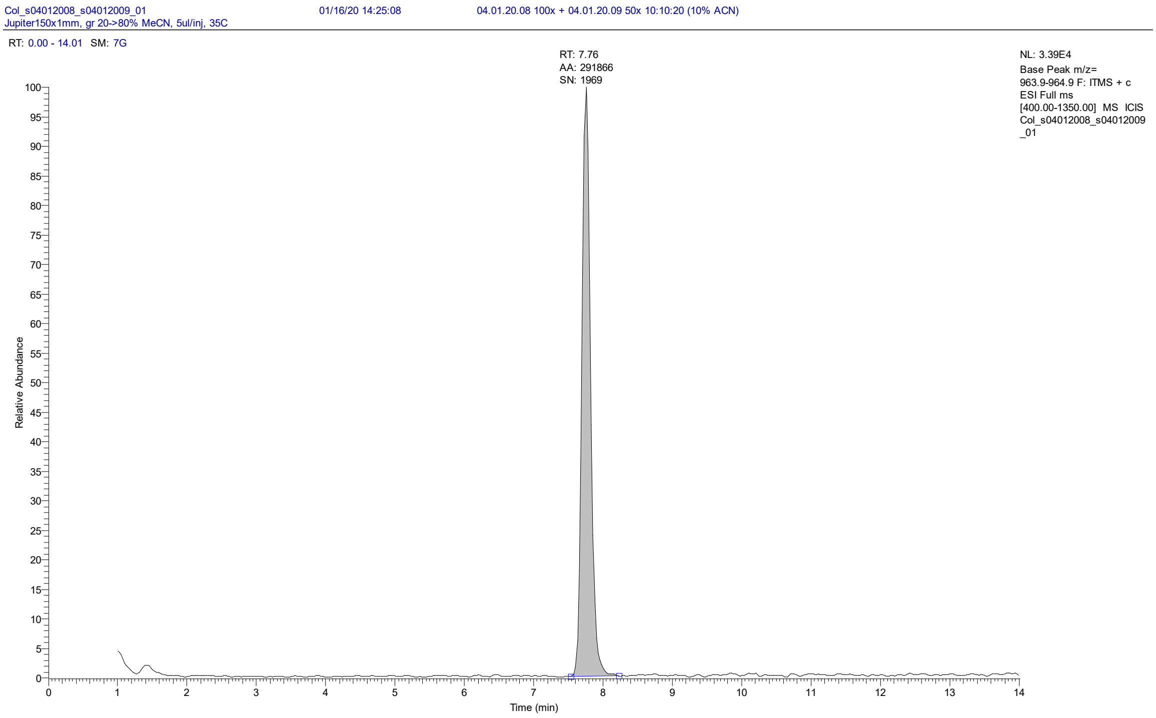

Fig. 2SF. A base peak LC-MS chromatogram of the synthetic AKH peptide Dircl-AKH-I pELTFSSGWG amide (MH^+ = 964.4).

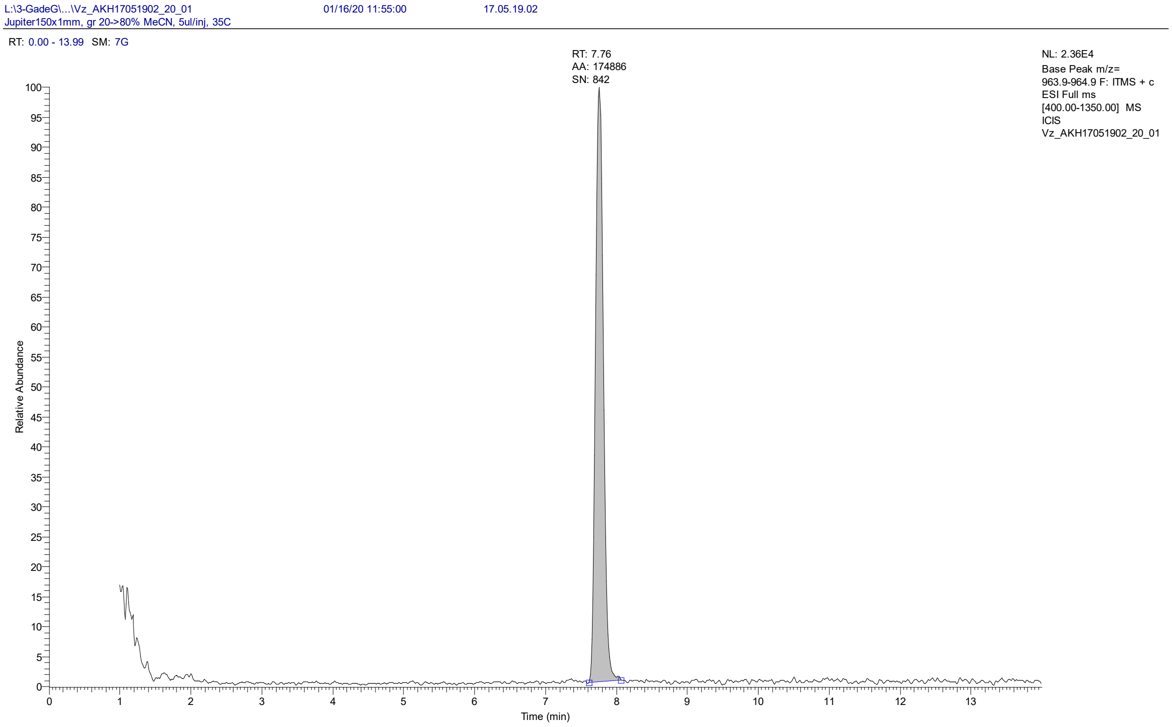

Fig. 2S G. A base peak LC-MS chromatogram of the detected AKH with MH^+ = 964.4 from the *D. clytus clytus* CC.

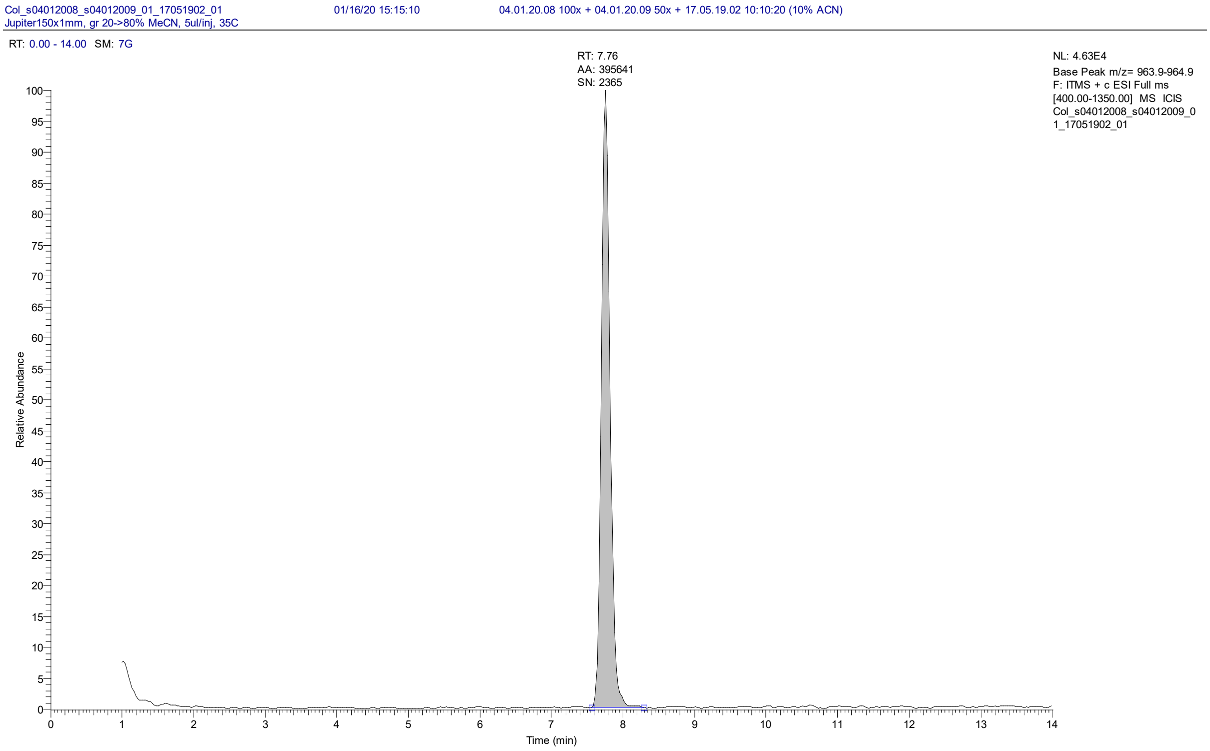

Fig. S2 H. A base peak LC-MS chromatogram of the detected AKH with MH^+ = 964.4 from the *D. clytus clytus* CC spiked with the synthetic Dircl-AKH-I peptide. The native peak co-elutes with the synthetic peptide and thus proves Leu at position 2.

Fig. S2 I-K. An LC-MS co-elution experiment of the CC extract-derived peptide with MH^+ 921.5 from the *D. clytus clytus* CC spiked with the synthetic peptide: pELTFSTGW amide (Dircl-AKH-II; MH^+ = 921.5). The chromatograms I-K reveal one prominent peak that co-incides with the retention time of the native peptide, thus indicating that the amino acid in position 2 is Leu, and not Ile.

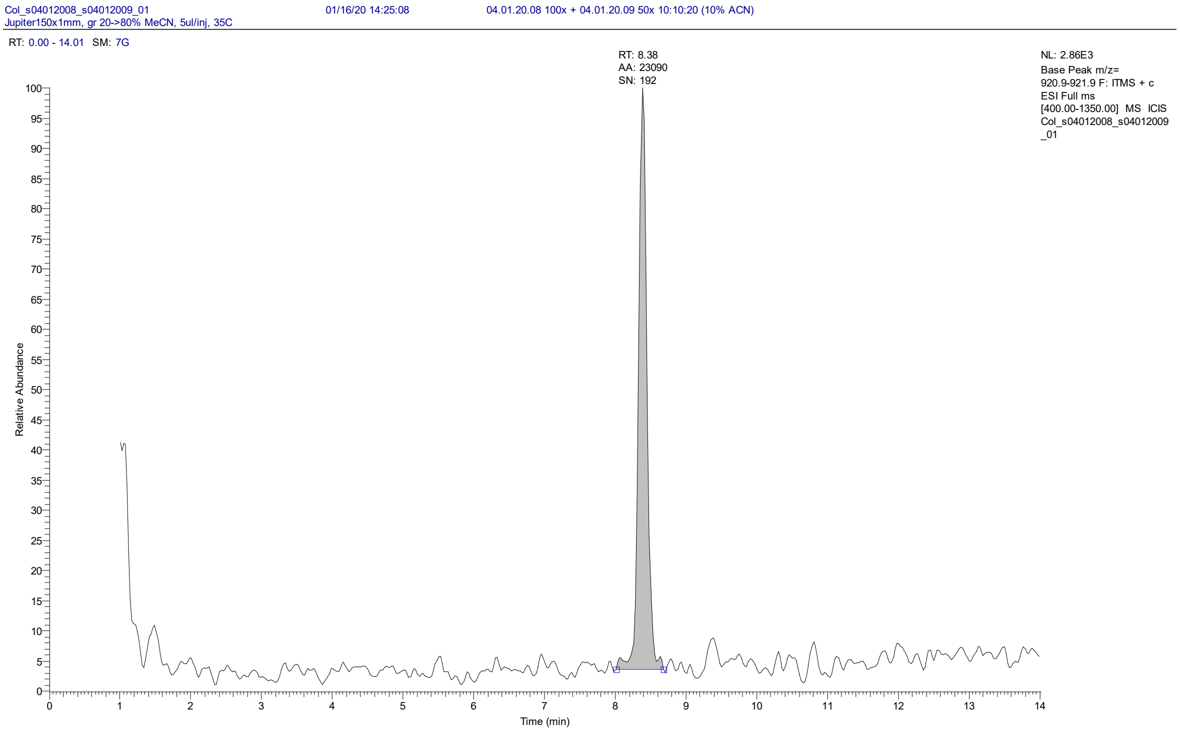

Fig. S2 I. A base peak LC-MS chromatogram of the synthetic AKH peptide Dircl-AKH-II pELTFSTGW amide (MH^+ = 921.5).

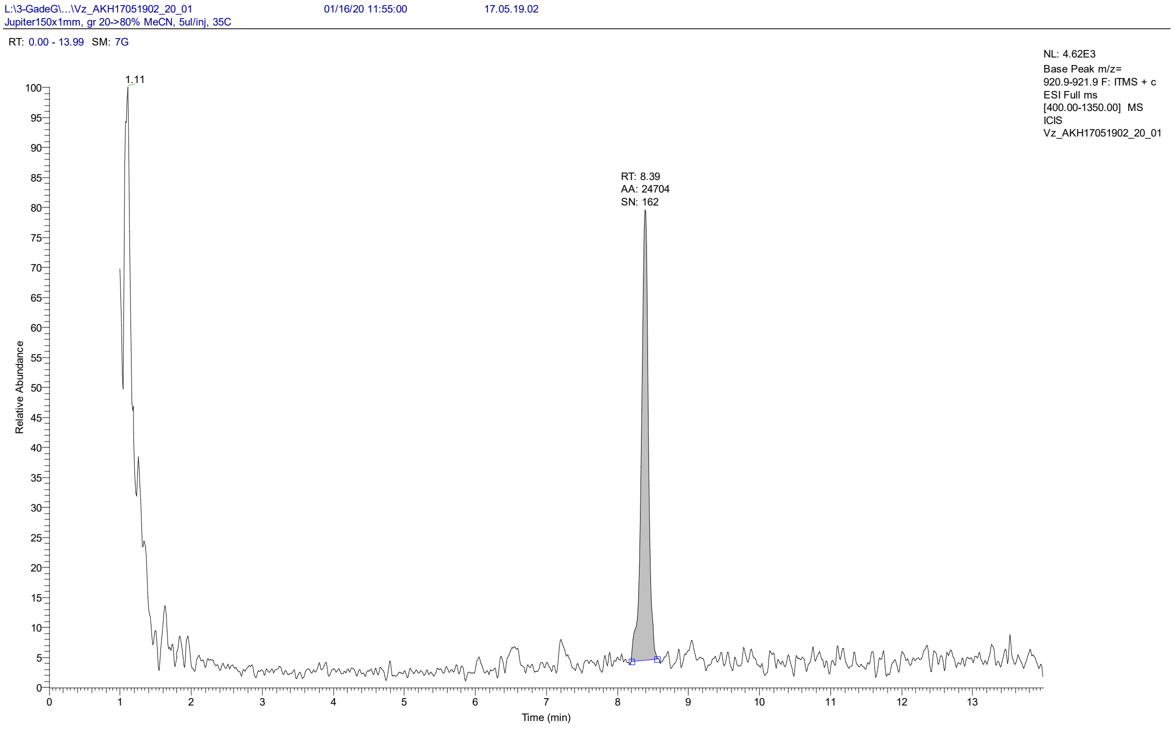

Fig. 2SJ. A base peak LC-MS chromatogram of the detected AKH with MH^+ = 921.5 from the *D. clytus clytus* CC.

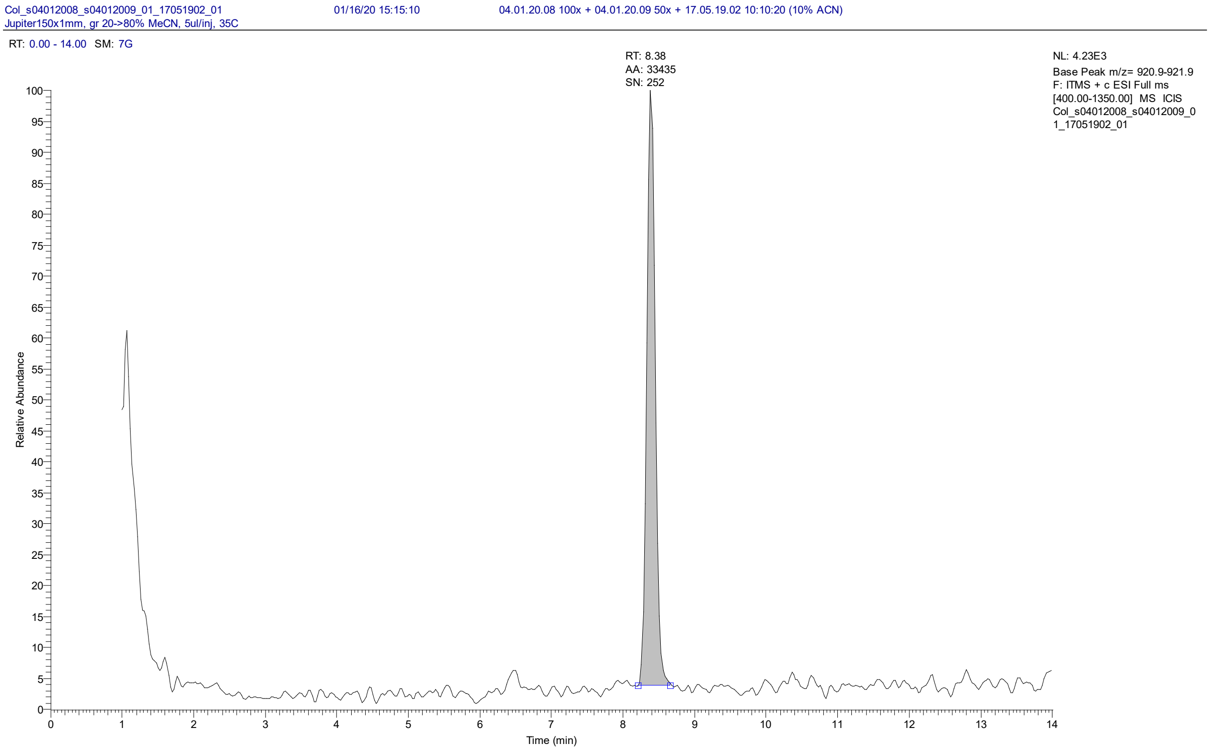

Fig. S2K. A base peak LC-MS chromatogram of the detected AKH with MH^+ = 921.5 from the *D. clytus clytus* CC spiked with the synthetic Dircl-AKH-II peptide. The native peak co-elutes with the synthetic peptide and thus proves Leu at position 2.

SUPPLEMENTARY FIGURE S3: Sequence elucidation and confirmation of the AKH peptide structures in the CC extract from *Acraea horta* by HPLC-MS co-elution of the native peptide peaks with the corresponding diluted synthetic AKHs.

Fig. S3 A-H. Base peak HPLC-MS chromatograms from the CC of *A. horta* showing the four detected AKH peptides.

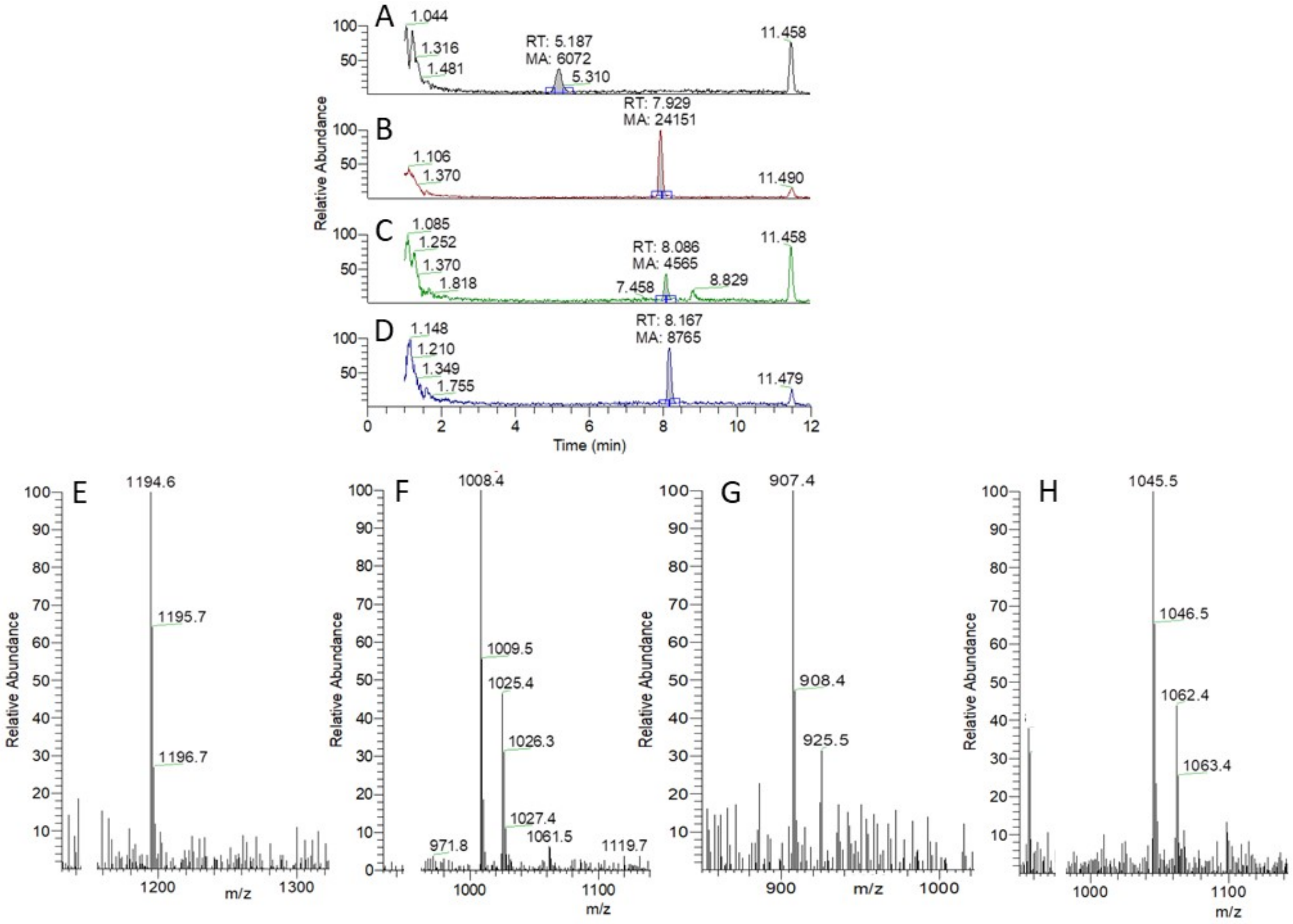

Fig. S3A -H. Base peak chromatograms obtained by LC-MS analysis showing the four detected AKH peptides in the CC extract of *A. horta* at retention times of 5.18 min (A) with MH^+ 1194.6 (E), 7.93 min (B) with MH^+ 1008.4 (F), 8.08 min (C) with MH^+ 907.4 (G) and 8.17 min with MH^+ 1045.5 (H).

Fig. S3 I and J. CID tandem MS+ESI spectra of the two ions with retention time at 8.08 and 8.17 min, respectively.

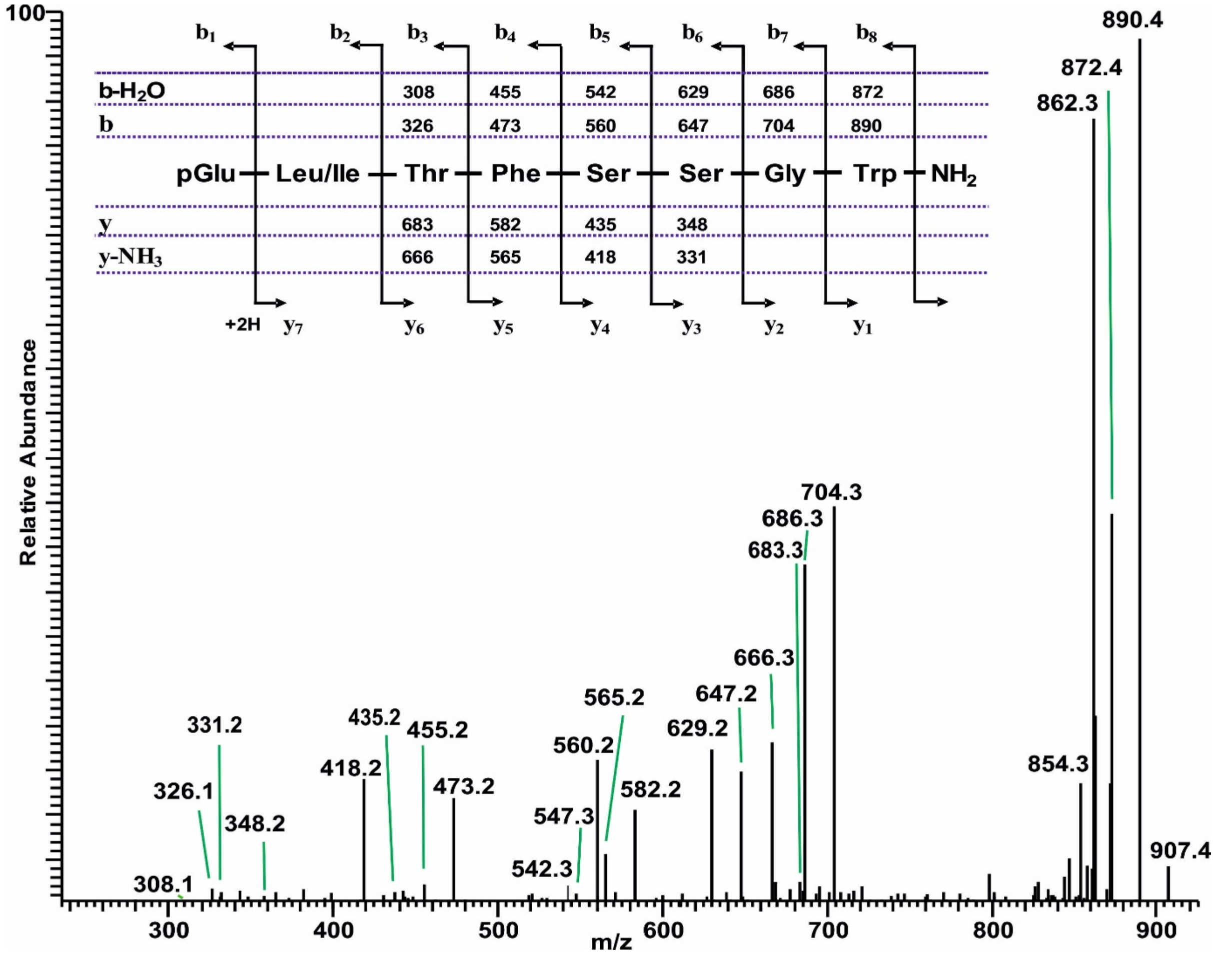

Fig. S3I. A CID tandem MS+ESI spectrum of the ion $[M + H]^+$ = 907.4 in Fig. S3G from the CC of *A. horta*.

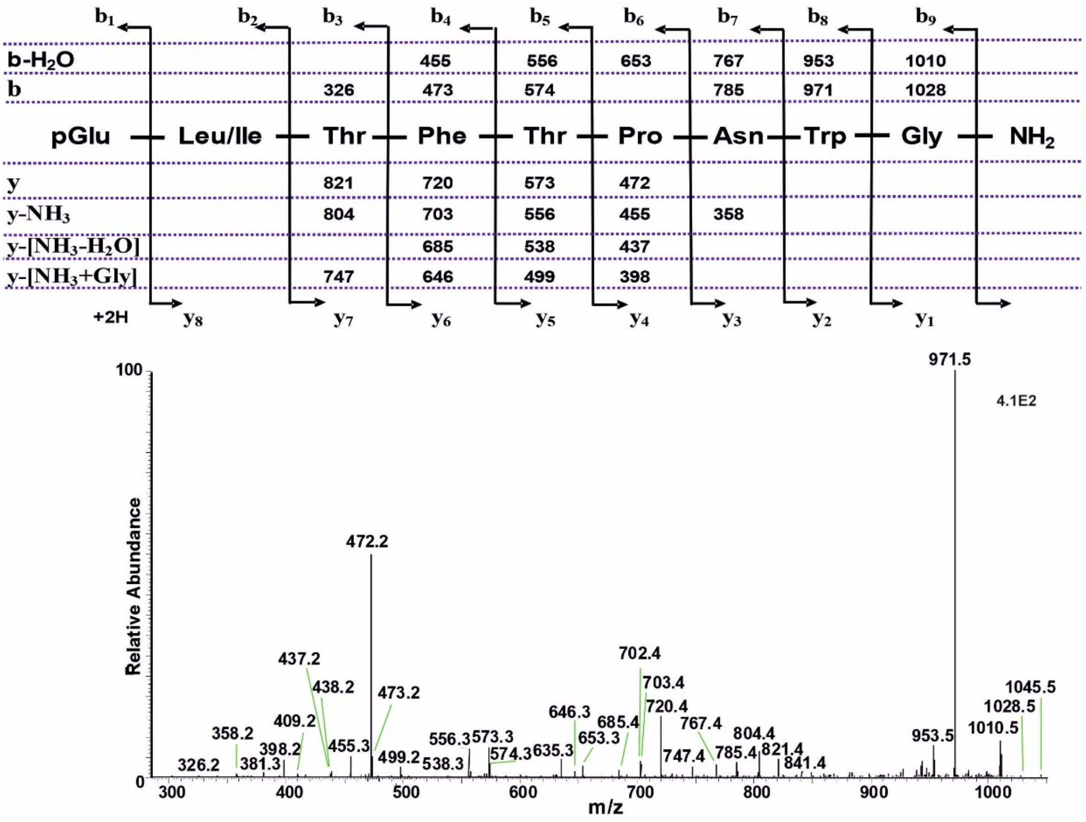

Fig. S3J. A CID tandem MS+ESI spectrum of the ion $[M + H]^+ = 1045.5$ in Fig. S3H from the CC of *A. horta*.

Fig. S3K. An LC-MS co-elution experiment of the CC extract from *A. horta* spiked with the synthetic peptides pELTFTSSWG amide (Manse-AKH; MH^+ = 1008.5), pELTFSSGW amide (Piebr-AKH; MH^+ = 907.5) and pELTFTPNWG amide (Triin-AKH; MH^+ = 1045.5).

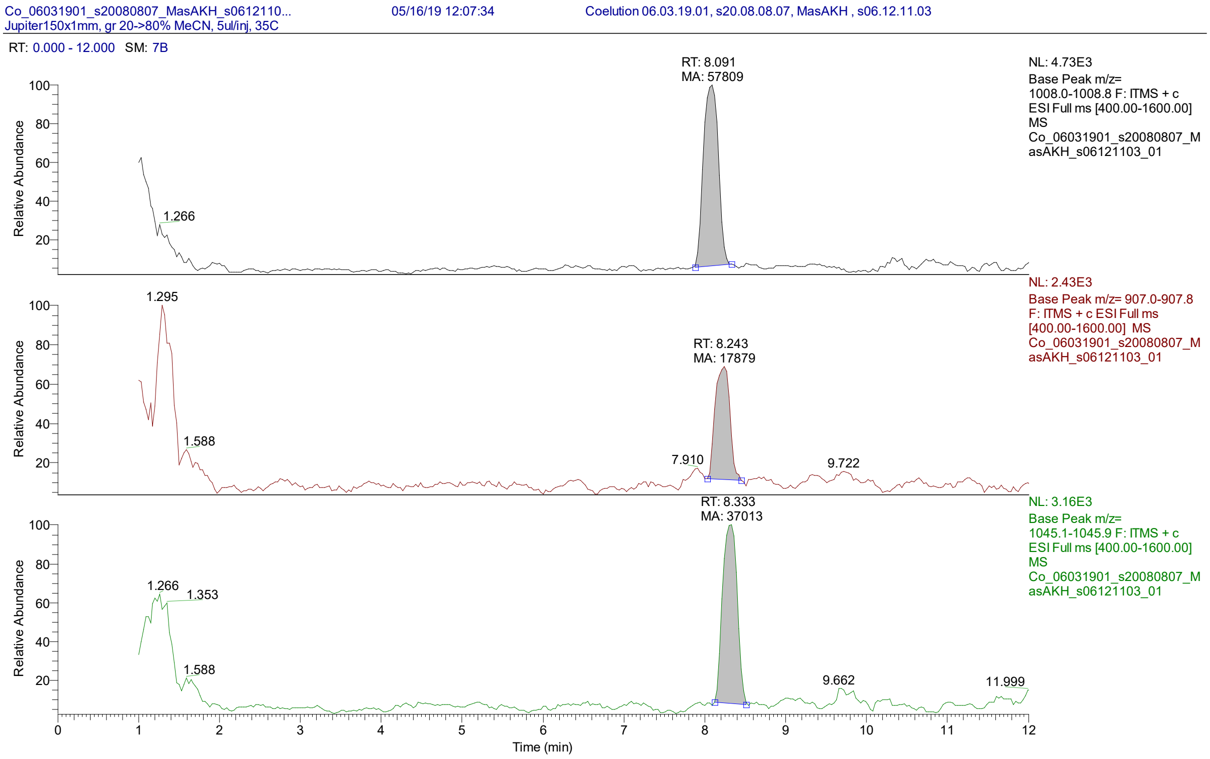

Fig. S3K. The extracted LC-MS chromatograms of the natural (CC extract) AKH peaks spiked with the synthetic AKHs: top panel shows Manse-AKH (RT = 8.09 min, MH^+ = 1008.4), middle panel shows Piebr-AKH ((RT = 8.23 min, MH^+ = 907.4); and the bottom panel shows Triin-AKH (RT = 8.33 min, MH^+ = 1045.5). The uniform chromatographic peaks confirmed the identity of the AKHs and the Leu at position 2 instead of Ile.

SUPPLEMENTARY FIGURE S4: Sequence elucidation and confirmation of the AKH peptide structures in the CC extract from *Chilo partellus* by HPLC-MS co-elution of the native peptide peaks with the corresponding diluted synthetic AKHs.

Fig. S4 A-E. Base peak HPLC-MS chromatograms from the CC of *C. partellus* showing the two detected AKH peptides.

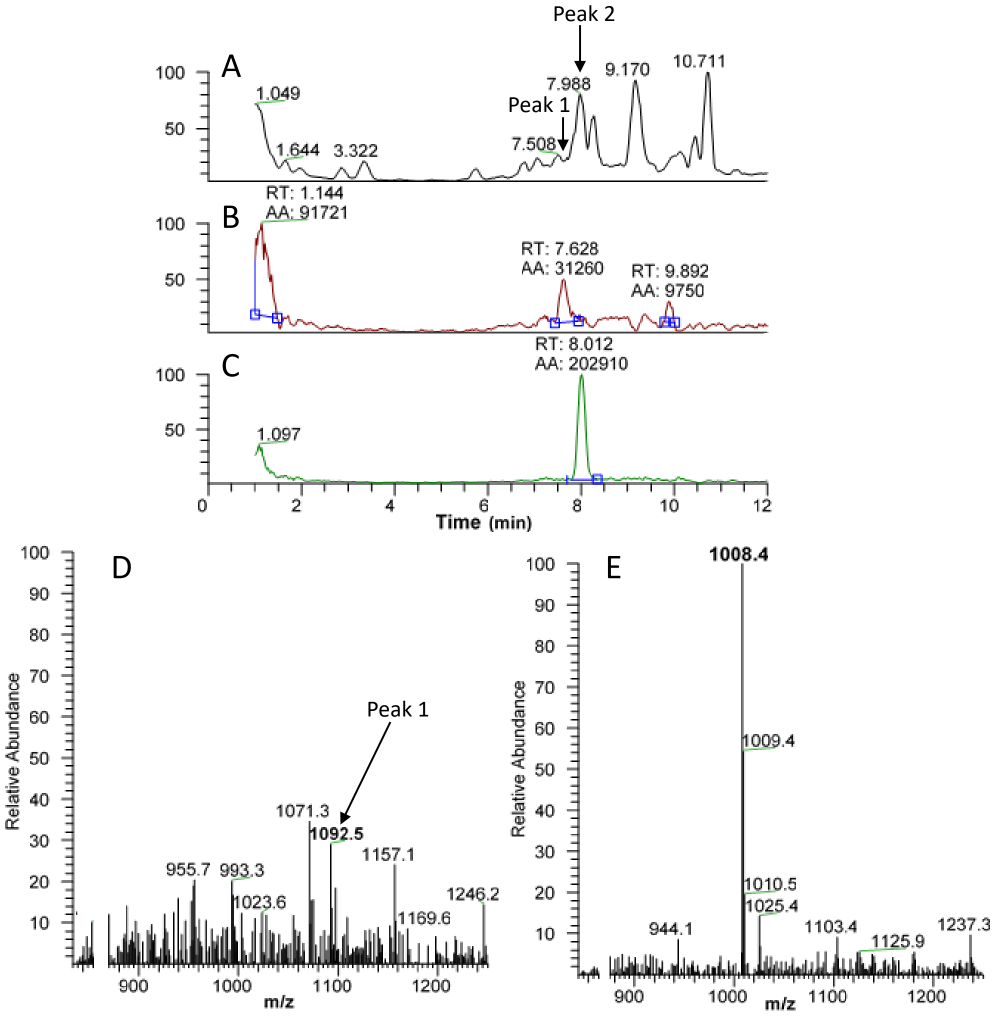

Fig. S4 A -E. (A) Base peak chromatogram obtained by LC-MS analysis showing detection of two AKH peptides labelled peak1 and peak 2 in the CC extract of *C. partellus*. **(B)** The extracted peak 1 at retention time of 7.62 min with MH^+ 1092.5 **(D),** and 8.01 min **(C)** with MH^+ 1008.4 **(E)**.

Fig. S4 F-H. An LC-MS co-elution experiment of the CC extract-derived peptide with MH$^+$ 1092.5 from the *C. partellus* CC spiked with the synthetic peptide pELTFSTGWGN amide (Chipa-AKH; MH$^+$ = 1092.5). The chromatograms F-H reveal one prominent peak that co-incides with the retention time of the native peptide, thus indicating that the amino acid in position 2 is Leu, and not Ile.

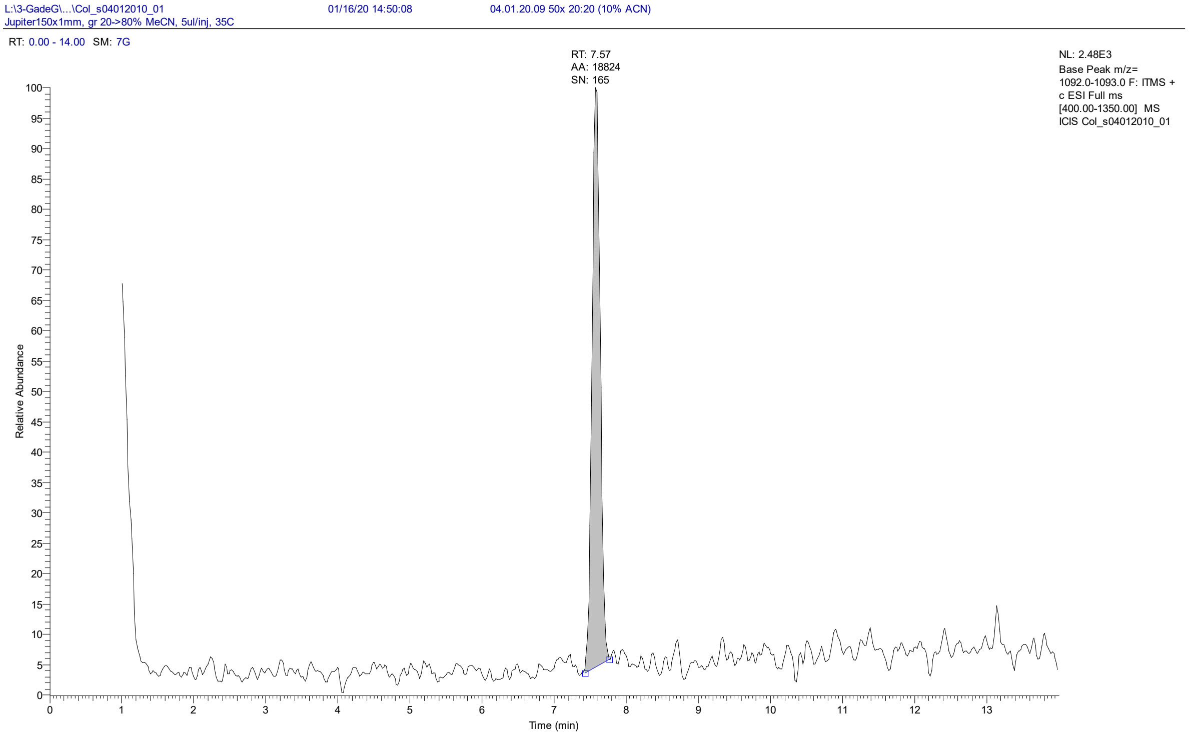

Fig. S4F. A base peak LC-MS chromatogram of the synthetic AKH peptide Chipa-AKH pELTFSTGWGN amide (MH$^+$ = 1092.5).

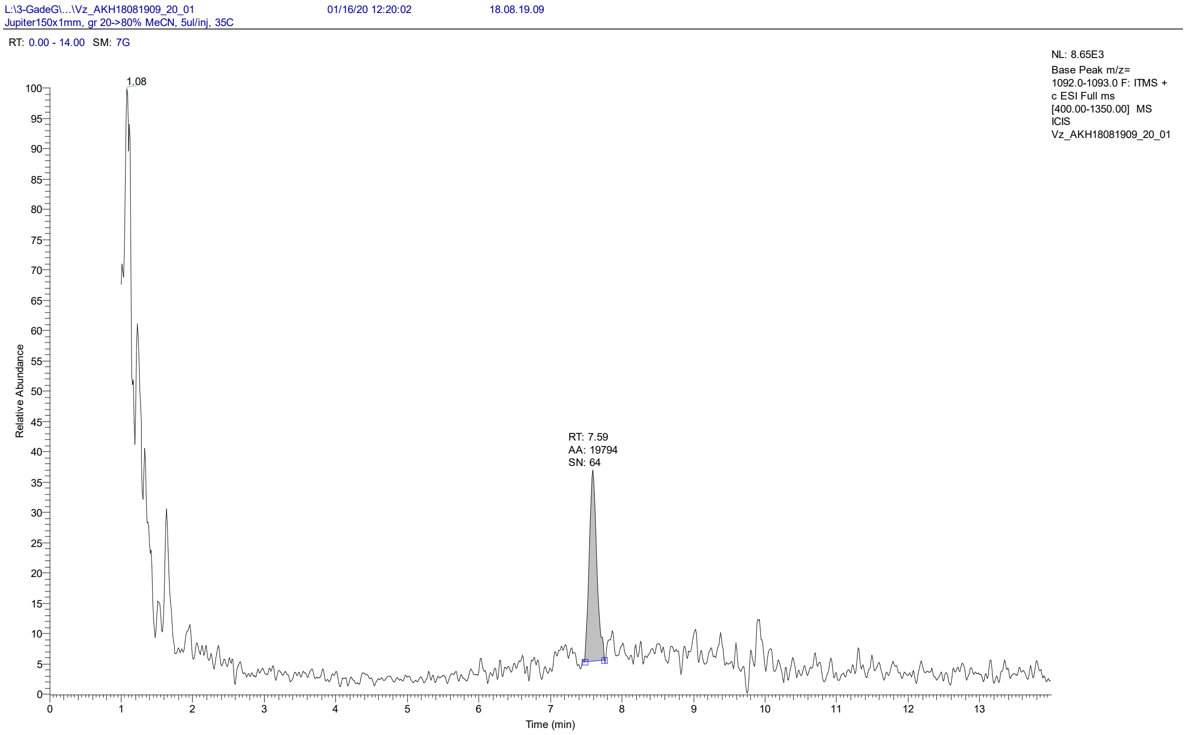

Fig. S4 G. A base peak LC-MS chromatogram of the detected AKH with MH^+ = 1092.5 from the *C. partellus* CC.

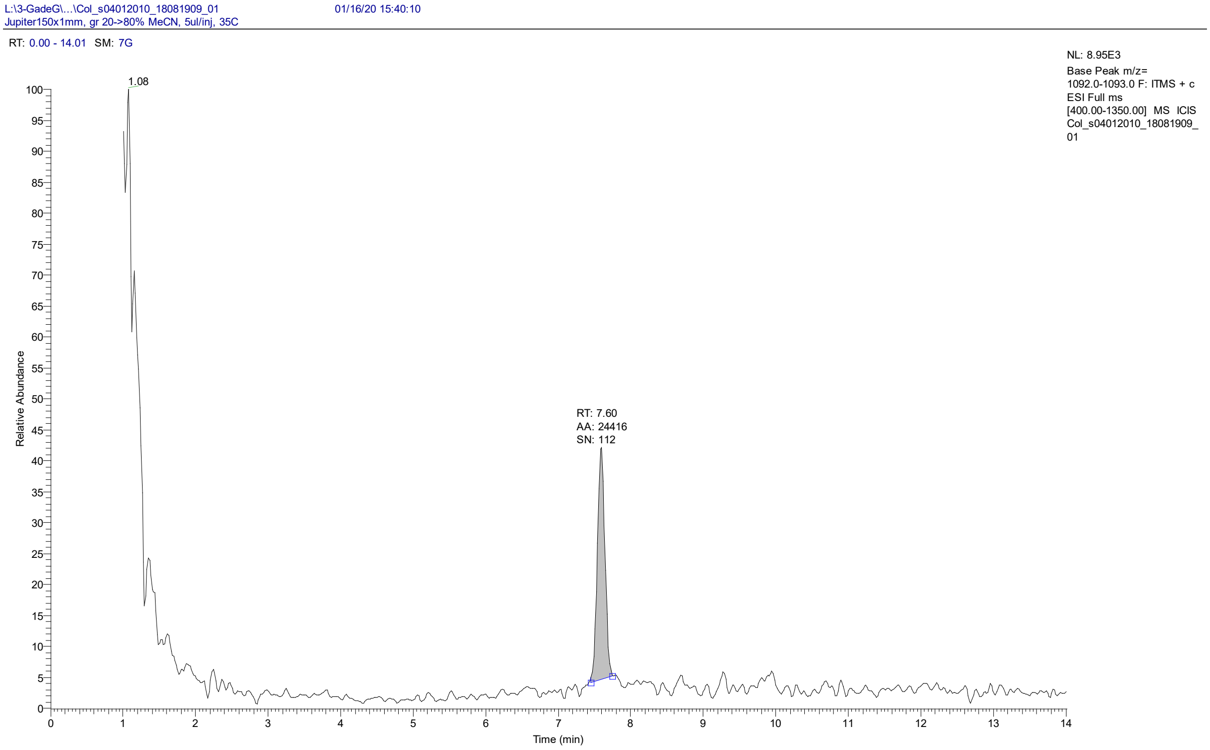

Fig. S4 H. A base peak LC-MS chromatogram of the detected AKH with MH^+ = 1092.5 from the *C. partellus* CC spiked with the synthetic Chipa-AKH peptide. The native peak co-elutes with the synthetic peptide and thus proves Leu at position 2.

SUPPLEMENTARY FIGURE S5: Sequence elucidation and confirmation of the AKH peptide structures in the CC extract from *Actias luna* by HPLC-MS co-elution of the native peptide peaks with the corresponding diluted synthetic AKHs.

Fig. S5 A-D. Base peak HPLC-MS chromatograms from the CC of *A. luna* showing the two detected AKH peptides.

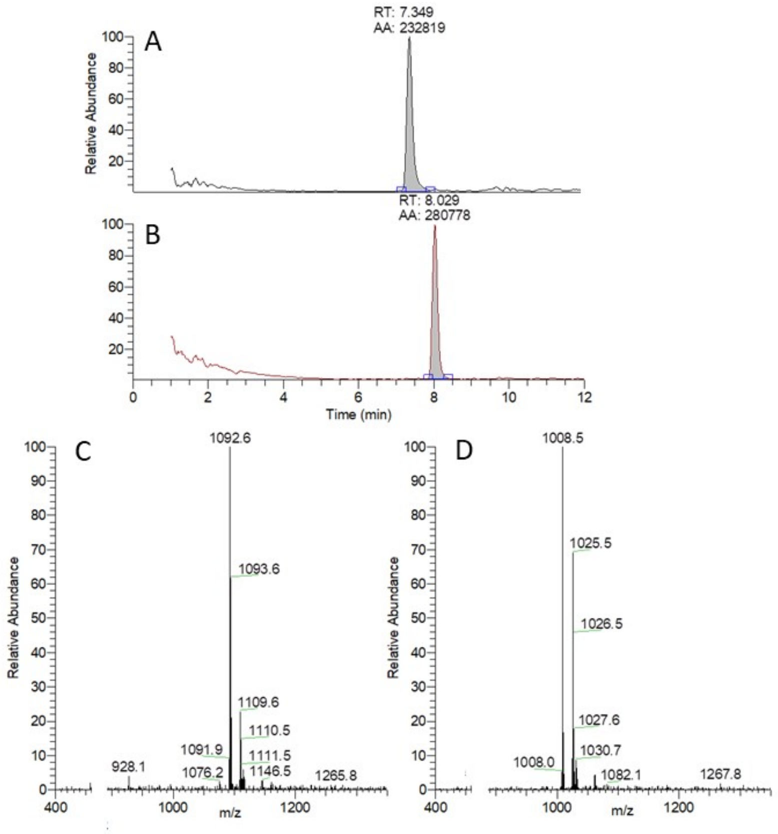

Fig. S5 A-D. Base peak chromatograms obtained by LC-MS analysis showing the two detected AKH peptides in the CC extract of *A. luna* at retention times of 7.35 min (A) with MH^+ 1092.6 (C) and 8.03 min (B) with MH^+ 1008.5 (D).

Fig. S5E. An LC-MS co-elution experiment of the CC extract from *A. luna* spiked with the synthetic peptides pELTFTSSWG amide (Manse-AKH; MH^+ = 1008.5) and pELTFSSGWGQ amide (Manse-AKH-II; MH^+ = 1092.5).

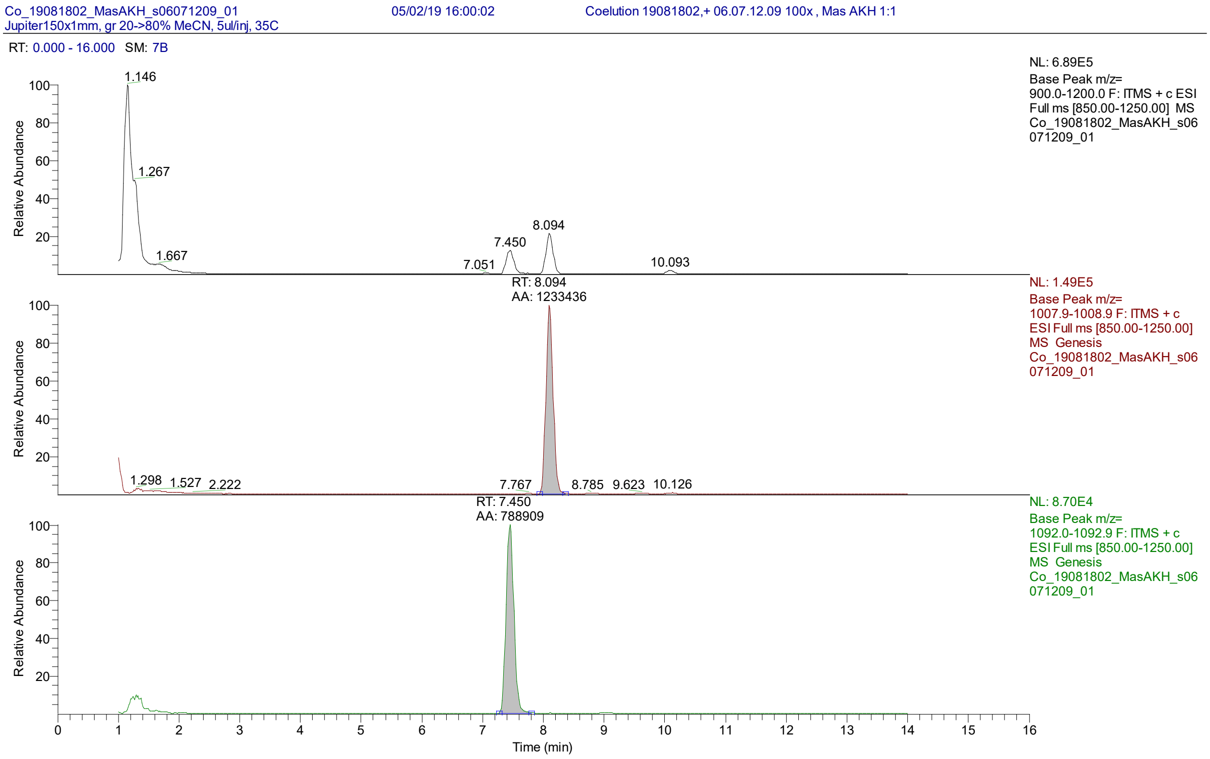

Fig. S5 E. Top. Base peak chromatogram obtained by LC-MS analysis of a CC extract from *A. luna* spiked with synthetic Manse-AKH and Manse-AKH-II. **Middle**: The extracted LC-MS chromatogram of Manse-AKH (MH^+ = 1008.4). **Bottom**: The extracted LC-MS chromatogram of Manse-AKH-II (MH^+ = 1092.5). The uniform peaks prove the presence of Leu at position 2 instead of Ile.

SUPPLEMENTARY FIGURE S6: Sequence elucidation and confirmation of the AKH peptide structures in the CC extract from *Antheraea yamamai* by HPLC-MS co-elution of the native peptide peaks with the corresponding diluted synthetic AKHs.

Fig. S6 A-C. Base peak HPLC-MS chromatograms from the CC of *A. yamamai* showing the two detected AKH peptides.

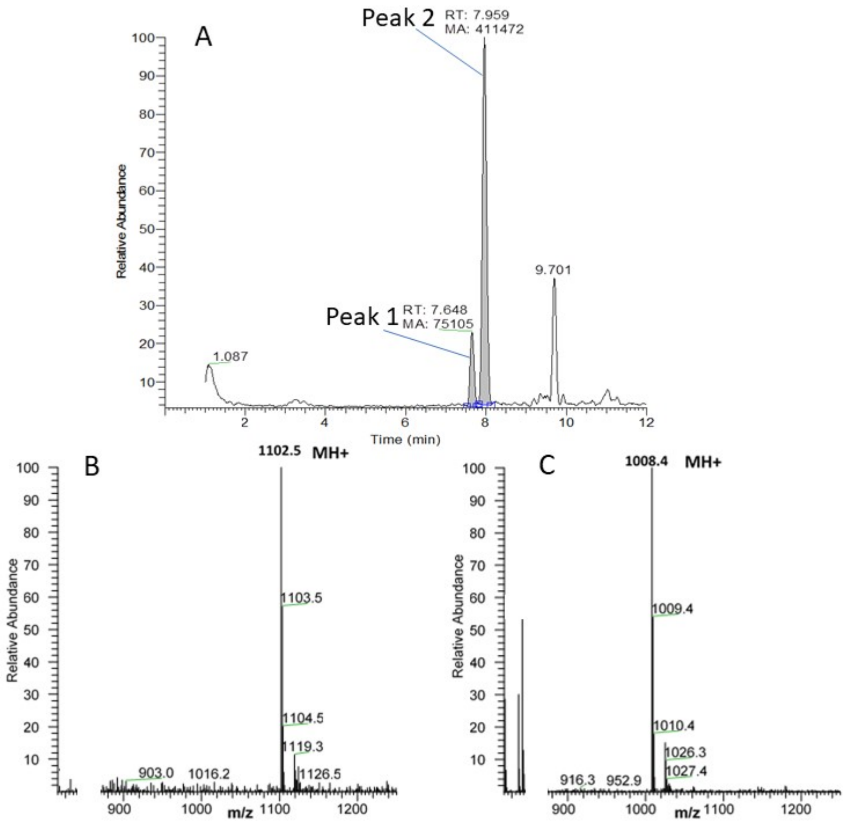

Fig. S6 A-C. Base peak chromatogram obtained by LC-MS analysis showing the two detected AKH peptides in the CC extract of *A. yamamai* at retention times of 7.64 min (A) with MH^+ 1102.5 (B) and 7.95 min with MH^+ 1008.4 (C).

Fig. S6 D-F. An LC-MS co-elution experiment of the CC extract-derived peptide with MH^+ 1008.4 from the *A. yamamai* CC spiked with the synthetic peptide: pELTFTSSWG amide (Manse-AKH; MH^+ = 1008.5). The chromatograms D-F reveal one prominent peak that co-incides with the retention time of the native peptide, thus indicating that the amino acid in position 2 is Leu, and not Ile.

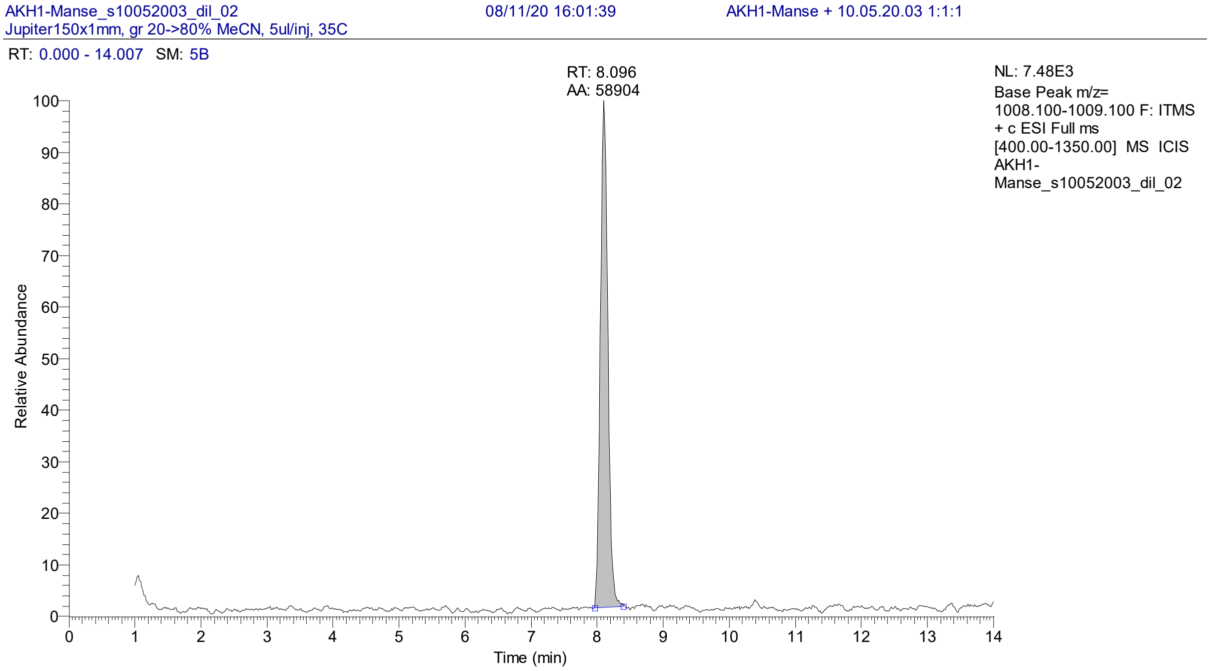

Fig. S6D. A base peak LC-MS chromatogram of the diluted synthetic Manse-AKH peptide pELTFTSSWG amide (MH^+ = 1008.5).

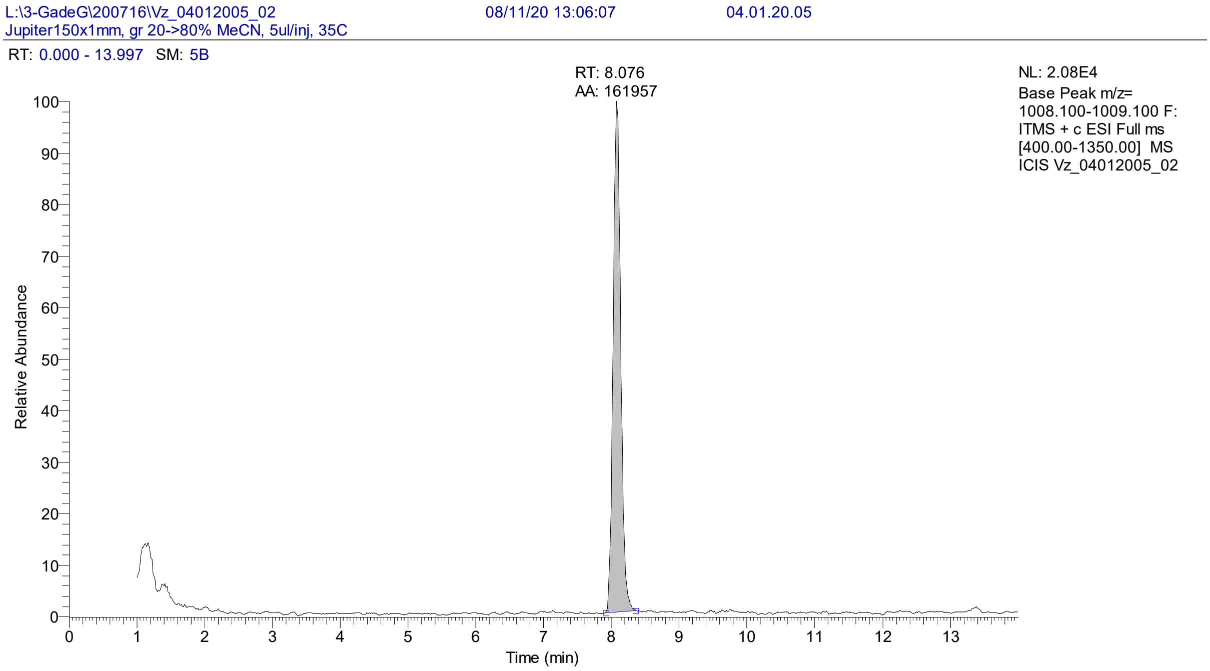

Fig. S6E. A base peak LC-MS chromatogram of the detected AKH with $MH^+ = 1008.5$ from the *A. yamamai* CC.

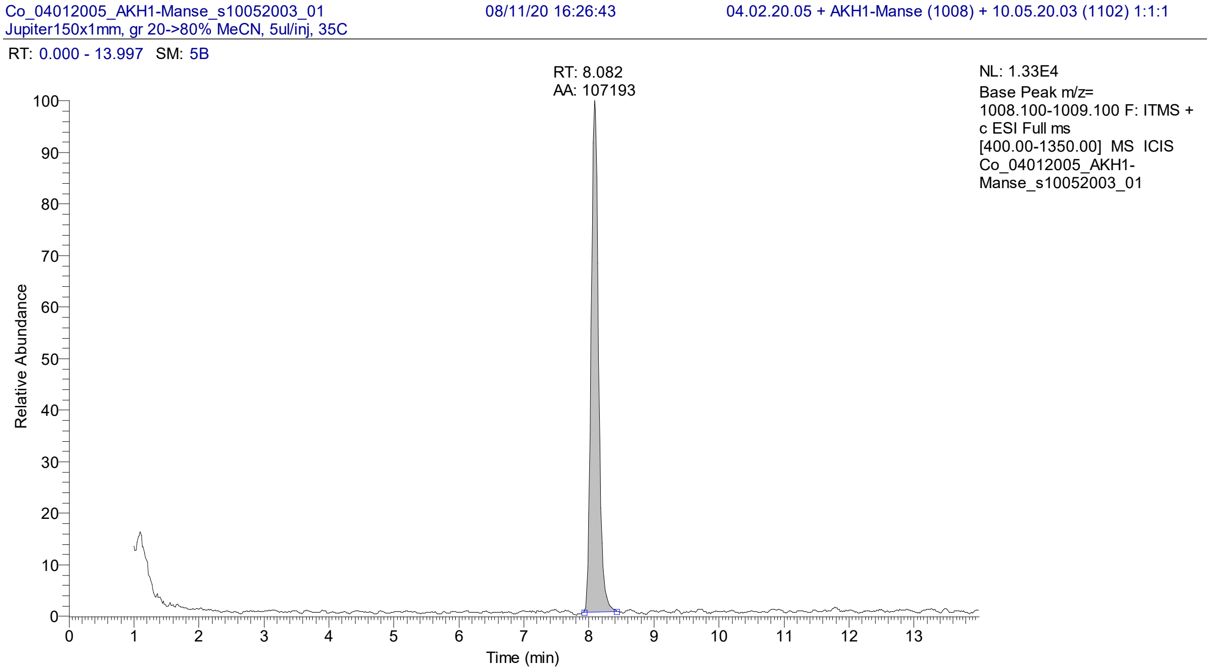

Fig. S6F. A base peak LC-MS chromatogram of the detected AKH with $MH^+ = 1008.5$ from the *A. yamamai* CC spiked with the synthetic Manse-AKH peptide. The native peak co-elutes with the synthetic peptide and thus proves Leu at position 2.

Fig. S6 G-I. An LC-MS co-elution experiment of the CC extract-derived peptide with MH^+ 1102.4 from the *A. yamamai* CC spiked with the synthetic peptide: pELTFSPGWGQ amide (Antya-AKH; MH^+ = 1102.5). The chromatograms G-I reveal one prominent peak that co-incides with the retention time of the native peptide, thus indicating that the amino acid in position 2 is Leu, and not Ile.

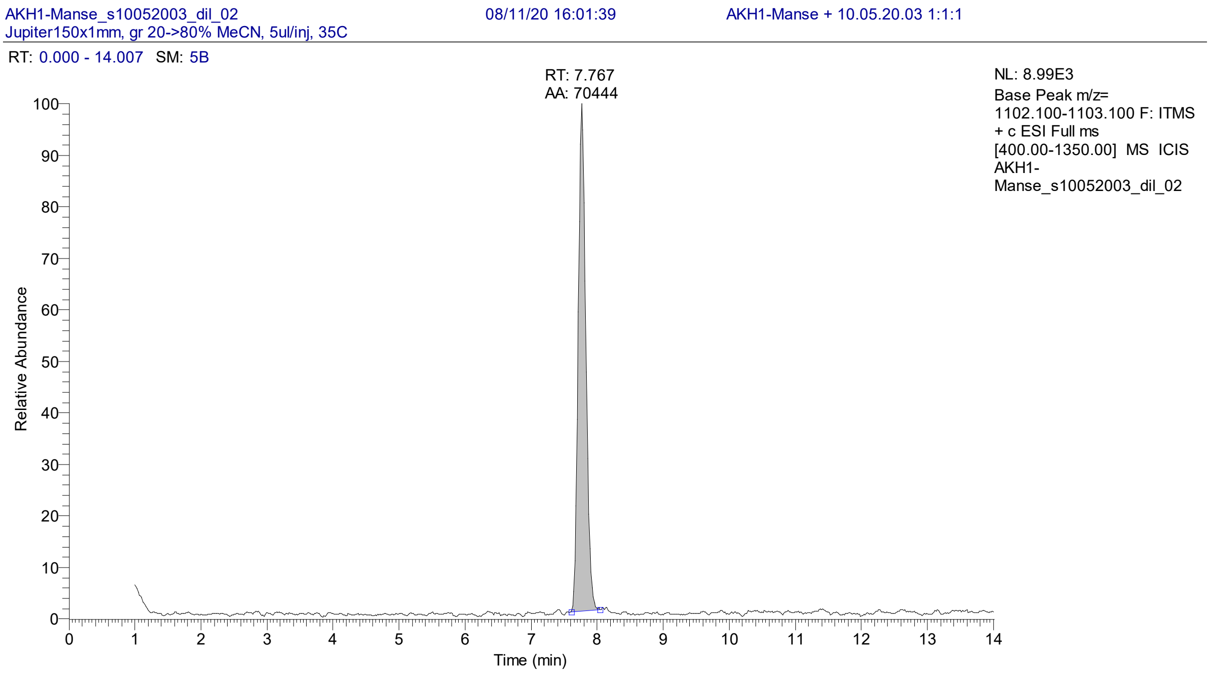

Fig. S6G. A base peak LC-MS chromatogram of the diluted synthetic Antya-AKH peptide pELTFSPGWGQ amide (MH^+ = 1102.5).

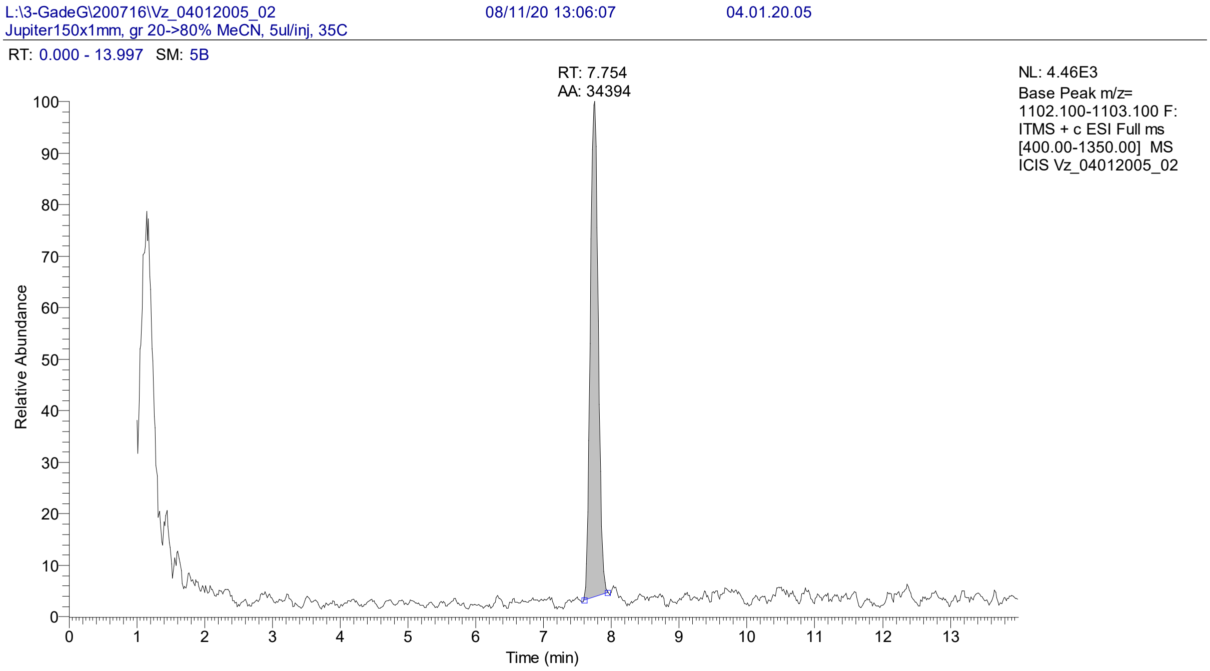

Fig. S6H. A base peak LC-MS chromatogram of the detected AKH with $MH^+ = 1102.5$ from the *A. yamamai* CC.

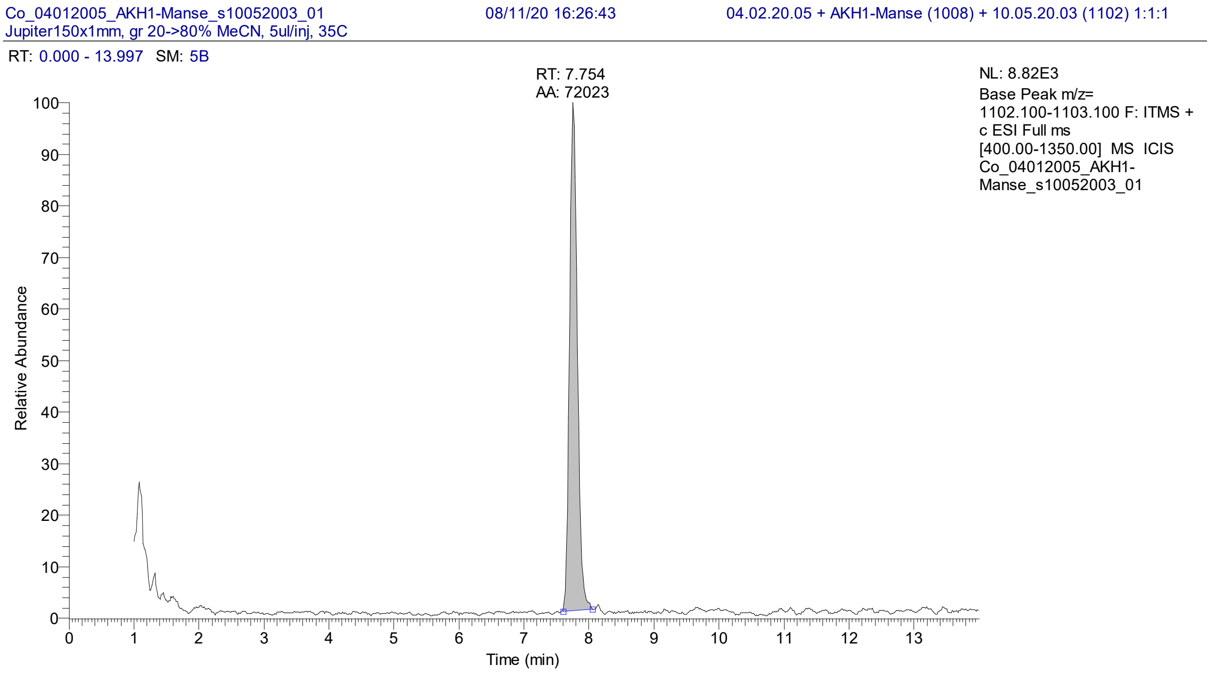

Fig. S6I. A base peak LC-MS chromatogram of the detected AKH with $MH^+ = 1102.5$ from the *A. yamamai* CC spiked with the synthetic Antya-AKH peptide. The native peak co-elutes with the synthetic peptide and thus proves Leu at position 2.
